# An efficient selenium transport pathway of selenoprotein P utilizing a high-affinity ApoER2 receptor variant and being independent of selenocysteine lyase

**DOI:** 10.1016/j.jbc.2023.105009

**Published:** 2023-07-03

**Authors:** Ayako Mizuno, Takashi Toyama, Atsuya Ichikawa, Naoko Sakai, Yuya Yoshioka, Yukina Nishito, Renya Toga, Hiroshi Amesaka, Takayuki Kaneko, Kotoko Arisawa, Ryouhei Tsutsumi, Yuichiro Mita, Shun-ichi Tanaka, Noriko Noguchi, Yoshiro Saito

**Affiliations:** 1 Laboratory of Molecular Biology and Metabolism, Graduate School of Pharmaceutical Sciences, Tohoku University, Sendai, Japan; 2 The Systems Life Sciences Laboratory, Department of Medical Life Systems, Faculty of Life and Medical Sciences, Doshisha University, Kyotanabe, Japan; 3 Laboratory of Biostructural Chemistry, Graduate School of Life and Environmental Sciences, Kyoto Prefectural University, Kyoto, Japan; 4 Department of Biotechnology, College of Life Sciences, Ritsumeikan University, Shiga, Japan

**Keywords:** selenium, selenoprotein P, ApoER2, *O*-linked sugar domain, lysosome, selenocysteine lyase

## Abstract

Selenoprotein P (SeP, encoded by the *SELENOP* gene) is a plasma protein that contains selenium in the form of selenocysteine residues (Sec, a cysteine analog containing selenium instead of sulfur). SeP functions for the transport of selenium to specific tissues in a receptor-dependent manner. Apolipoprotein E receptor 2 (ApoER2) has been identified as a SeP receptor. However, diverse variants of ApoER2 have been reported, and the details of its tissue specificity and the molecular mechanism of its efficiency remain unclear. In the present study, we found that human T lymphoma Jurkat cells have a high ability to utilize selenium *via* SeP, while this ability was low in human rhabdomyosarcoma cells. We identified an ApoER2 variant with a high affinity for SeP in Jurkat cells. This variant had a dissociation constant value of 0.67 nM and a highly glycosylated *O*-linked sugar domain. Moreover, the acidification of intracellular vesicles was necessary for selenium transport *via* SeP in both cell types. In rhabdomyosarcoma cells, SeP underwent proteolytic degradation in lysosomes and transported selenium in a Sec lyase–dependent manner. However, in Jurkat cells, SeP transported selenium in Sec lyase–independent manner. These findings indicate a preferential selenium transport pathway involving SeP and high-affinity ApoER2 in a Sec lyase–independent manner. Herein, we provide a novel dynamic transport pathway for selenium *via* SeP.

The essential trace element selenium (Se) is very reactive and has potent toxicity. However, the living body can use its reactivity for redox reactions (1, 2). The biological function of Se is mainly mediated by selenoproteins. These proteins consist of selenocysteine (Sec), which is a cysteine analog that contains Se instead of sulfur. Twenty-five types of human selenoproteins have been identified, and they play diverse roles in the maintenance of redox homeostasis in the human body (3, 4). Glutathione peroxidase (GPx), which is a representative selenoprotein, is involved in the reduction of hydroperoxide, while thioredoxin reductase, another selenoprotein, functions for redox regulation (5). Selenoprotein P (SeP), which is encoded by the *SELENOP* gene, is a major selenoprotein in the plasma and is mainly synthesized in the liver and secreted into the plasma (6, 7). The “P” in SeP denotes its presence in plasma. SeP is a unique selenoprotein that possesses 10 Sec residues, while most selenoproteins have only one Sec residue. Multiple Sec residues in SeP are important for its function; one N-terminal Sec residue forms an active site with GPx-like activity to reduce phospholipid hydroperoxide, while the nine C-terminal Sec residues function as a Se transporter (8, 9, 10). The biological significance of the effective Se transport system involving SeP has been demonstrated using *in vitro* and *in vivo* experiments. A decrease in the tissue Se levels in the brain, kidney, testis, and bone of SeP KO mice has been reported (11, 12, 13, 14). A decrease in SeP levels causes a deficiency of selenoproteins and various dysfunctions along with oxidative stress (15), while excessive SeP levels induce insulin resistance, which leads to type 2 diabetes (16). In patients with type 2 diabetes, levels of SeP mRNA in the liver and SeP protein in the blood were increased (16, 17). Moreover, excessive SeP levels impaired insulin resistance in the skeletal muscle and liver and the insulin secretory capacity of pancreatic β cells (18). Excessive SeP levels increased exercise resistance and impaired cold-induced thermogenesis in brown fat, indicating systematic deterioration effects on glucose and energy metabolism (19, 20). Thus, excessive SeP levels are a significant target for the treatment of lifestyle-related diseases (21). The human body exhibits a U-shaped reaction against SeP levels, and SeP expression and Se transport *via* SeP are strictly controlled, but the details remain unclear.

The Se transport system *via* SeP is mediated by its receptors. Three kinds of low-density lipoprotein receptor–related proteins (LRPs) have been identified as SeP receptors, namely, apolipoprotein E receptor 2 (ApoER2/LRP8), megalin (LRP2), and LRP1 (19, 22, 23). Based on the phenotype of mice with gene KOs for each of these receptors, ApoER2 has been associated with SeP uptake in the brain, testis, and bone; megalin with the uptake in the kidney and brain; and LRP1 with the uptake in the skeletal muscle. A decrease in Se levels in the brain and testis of *SELENOP* ^Δ240-361^ mice, in which the Sec-rich C-terminal domain of SeP had been deleted, has been reported (24).

ApoER2, which is a single transmembrane protein, has a domain structure composed of ligand binding–type repeats, epidermal growth factor–like repeats, a YWTD β-propeller domain, an *O*-linked sugar domain, a transmembrane domain, and a cytoplasmic domain (25, 26). ApoE, which is involved in transporting fatty acids to tissues, and Reelin, which is involved in fetal brain development, are the major ligands other than SeP that bind to the ligand binding–type repeats of ApoER2 (27, 28). In the case of SeP, the C-terminal Sec-rich domain interacts with the YWTD β-propeller domain of ApoER2 (29). Diverse transcript variants of ApoER2 have been found (26). However, the knowledge of the relationship between ApoER2 variants and SeP uptake is limited, and the details are still unknown.

The Se in SeP is genetically encoded as Sec in the polypeptide chain and is covalently bonded to amino acid-backbone as its side chain (7, 29). Thus, to use Se of SeP for cellular selenoprotein synthesis, several biochemical steps are necessary. Moreover, SeP is incorporated into the cell and is then degraded in the lysosome. Sec that is generated *via* this process is cleaved by Sec lyase, leading to the formation of hydrogen selenide (H_2_Se), which is further phosphorylated to H_2_SePO_3_ by selenophosphate synthetase 2 (30, 31, 32). Finally, Sec is synthesized on transfer ribonucleic acid. Furthermore, mice lacking Sec lyase do not have Se-deficient phenotypes, such as male sterility, suggesting that the unidentified pathway of the Se transport system *via* SeP is independent of Sec lyase (33, 34).

In the present study using several types of cultured cells, we investigated the highly effective Se transport system of Jurkat cells and found the involvement of the high-affinity ApoER2 variant possessing the *O*-linked sugar domain. We also found that the Se transport occurs in a Sec lyase–independent manner. Herein, we provide the novel molecular mechanisms of a new and high-efficiency Se transport system *via* SeP.

## Results

### Different Se-supply activities of SeP depending on cells

**Figure 1:**
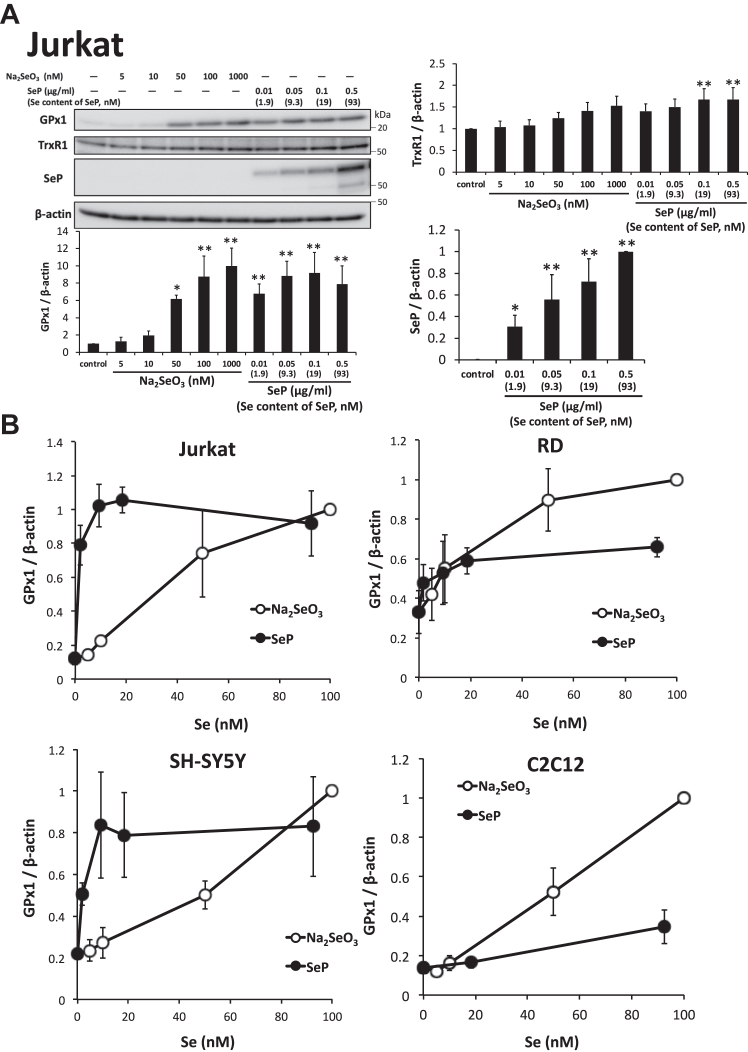
**Different Se-supply activities of SeP depending on the cell types.***A*, cellular uptake and Se-supply activity of SeP in Jurkat cells. Se-deficient Jurkat cells were treated with different concentrations of sodium selenite (Na_2_SeO_3_) and purified human SeP protein for 24 h. Then, whole cell lysates were subjected to Western blotting using anti-GPx1 Ab, anti-TrxR1 Ab KB12, and anti-SeP Ab BD1. GPx1, TrxR1, and SeP levels in the whole cell lysates were determined using Western blotting (n = 3, means ± s.d.). The band intensities of SeP were only evaluated in SeP-treated cells. The selenium content of SeP was determined, and purified SeP with 185 nmol Se/mg of protein was used in these experiments. *∗∗p* < 0.01, *∗p* < 0.05, *versus* control, Tukey ANOVA. *B*, comparison of Se-supply activity of SeP in each cell line. Relative GPx1 levels in Na_2_SeO_3_- or SeP-treated cells were plotted against the selenium concentration of each condition. GPx, glutathione peroxidase; SeP, selenoprotein P; Se, selenium.

The Se-supply activity of SeP was compared with that of sodium selenite in cultured cells. Se-deficient human T lymphoma Jurkat cells were cultured using different concentrations of purified human SeP and sodium selenite, and concentration-dependent increases in GPx1 and TrxR1 levels were observed (Fig. 1*A*). The increase in GPx1 levels was evident, and a significant increase was observed from SeP concentrations of 0.01 μg/ml (Fig. 1*A*). The change in TrxR1 levels was marginal and was significant only at SeP concentrations of 0.1 and 0.5 μg/ml (Fig. 1*A*). Moreover, a concentration-dependent increase in the specific SeP band was observed (Fig. 1*A*). These findings suggest that GPx1 and SeP levels are suitable markers for the Se-supply activity of SeP. In the case of mouse C2C12 myocytes, GPx1 and SeP levels significantly increased in a selenite- and SeP concentration-dependent manner, while TrxR1 levels were not significantly increased (Fig. S1*A*). The efficacy of SeP was completely different in different cell types. SeP concentrations of 0.05 μg/ml and 5 μg/ml were required to induce maximum GPx1 levels in Jurkat cells and C2C12 myocytes, respectively (Figs. 1*A* and S1*A*). In the case of human rhabdomyosarcoma (RD) cells, the efficacy of SeP was low (Fig. S1*B*), while in human neuroblastoma SH-SY5Y cells, SeP exhibited high Se-supply activity (Fig. S1*C*). GPx1 levels in each cell type treated with sodium selenite or SeP were plotted against the Se concentrations (Fig. 1*B*). These findings clearly indicated that the efficacy of sodium selenite was similar in these cells, but the Se-supply activity of SeP was different depending on the cell type (Figs. 1*B* and S2*A*).

### Analysis of cell surface binding and Se-supply activity of SeP in Jurkat cells using ^75^Se-labeled SeP

**Figure 2:**
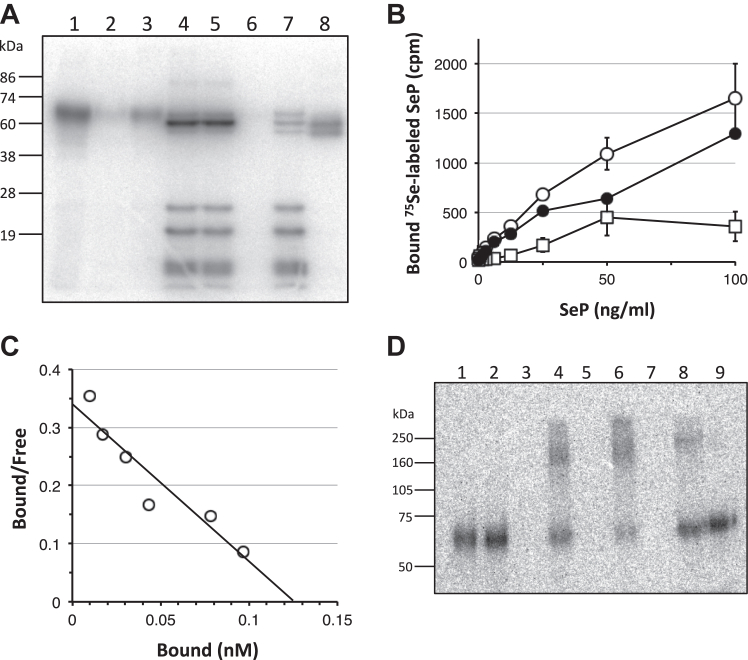
**Analysis of binding and Se-supply activity of SeP in Jurkat cells using**^**75**^**Se-labeled SeP.***A*, immunoprecipitation of ^75^Se-labeled SeP and cytosol from the cells cultured with ^75^Se-labeled SeP. ^75^Se-labeled SeP prepared from HepG2 cells was subjected to SDS-PAGE and analyzed using autoradiography (lane 1). Control Ab-conjugated beads (lane 2) or anti-SeP mAb-conjugated beads (lane 3) were treated with ^75^Se-labeled SeP, and bound proteins were subjected to SDS-PAGE and analyzed as described above. Cytosol from Jurkat cells that were cultured in the presence of ^75^Se-labeled SeP for 72 h was analyzed (lane 4). Anti-SeP mAb-conjugated beads (lanes 5 and 6) and anti-TrxR1 mAb KB12-conjugated beads (lanes 7 and 8) were added to the above cytosol, and its supernatants (lanes 5 and 7) and bound proteins (lanes 6 and 8) were subjected to SDS-PAGE. *B*, binding of ^75^Se-labeled SeP to Jurkat cells. The cells were treated with ^75^Se-labeled SeP in the absence (total binding, *open circles*) or presence (nonspecific binding, open squares) of a 500-fold excess of unlabeled SeP for 1 h at 4 °C. The specific binding (*closed circles*) was calculated from the difference between the total and nonspecific binding. *C*, the specific binding of ^75^Se-labeled SeP was analyzed using a Scatchard plot. The representative result is shown. Based on independent analysis (n = 4), a Kd value of 0.67 ± 0.33 nM and a number of binding sites of 2200 ± 1500 sites per cell were obtained. *D*, ^75^Se-labeled SeP and its binding protein complex were detected using chemical cross-linking. The cells were incubated with ^75^Se-labeled SeP in the absence (lanes 2, 4, 6, 8, and 9) or presence (lanes 3, 5, and 7) of a 500-fold excess of unlabeled SeP and then cross-linked, solubilized, and subjected to SDS-PAGE under nonreducing (lanes 2–7) or reducing (lanes 8 and 9) conditions. Lane 1, ^75^Se-labeled SeP (nonreducing condition); lanes 2 and 3, DMSO control; lanes 4, 5, and 8, disuccinimidyl suberate as a cross-linker; lanes 6, 7 and 9, dithiobis (succinimidyl propionate) as a cross-linker. SeP, selenoprotein P; Se, selenium.

Next, we analyzed cell surface binding and Se-supply activity of SeP using ^75^Se-labeled SeP in Jurkat cells, which showed the highest affinity for SeP in the cultured cells used. The purified ^75^Se-labeled SeP showed a single band at 69 kDa (Fig. 2*A*, lane 1). Immunoprecipitation of radiolabeled SeP using immobilized anti-SeP Ab resulted in a total loss of the 69-kDa band (Fig. 2*A*, lane 2), and this band was recovered in the precipitants (Fig. 2*A*, lane 3). To confirm the direct incorporation of Se derived from SeP into cellular selenoproteins, Jurkat cells were cultured for 72 h with ^75^Se-labeled SeP. Several radiolabeled selenoproteins were detected in the whole cell lysates using autoradiography (Fig. 2*A*, lane 4). These proteins were not affected when immobilized anti-human SeP Ab was added (Fig. 2*A*, lane 5), and ^75^Se-labeled band was not detected in its precipitants (Fig. 2*A*, lane 6), while immobilized anti-human TrxR1 Ab decreased the 60-kDa band and this band was recovered in the precipitants (Fig. 2*A*, lanes 7 and 8).

We further performed a binding assay using ^75^Se-labeled SeP. The binding of ^75^Se-labeled SeP to Jurkat cells was observed with increasing amounts of the radiolabeled protein at 4 °C for 1 h (Fig. 2*B*). We also found the inhibitory effect of unlabeled SeP, and the specific binding was estimated from the difference between the total and nonspecific binding. The affinity of SeP to Jurkat cells was determined using the Scatchard analysis. The Scatchard plots showed linearity (Fig. 2*C*). The dissociation constant (Kd) was calculated as 0.67 ± 0.33 nM, and the number of binding sites was 2200 ± 1500 per cell (n = 4). Binding assays were also performed to determine the amount of binding of ^75^Se-labeled SeP to Se-deficient Jurkat cells, and an essentially identical number of binding sites with the same affinity was observed (data not shown).

A cross-linking study was performed to define the size of the surface molecule on Jurkat cells that was responsible for SeP binding. When Jurkat cells were incubated with ^75^Se-labeled SeP (50 ng/ml), covalently cross-linked using 1 mM disuccinimidyl suberate, and analyzed using SDS-PAGE under nonreducing conditions, a ^75^Se-labeled broad band of approximately 200 kDa was observed (Fig. 2*D*, lane 4). In the absence of a cross-linker, SeP in the cell lysate migrated to the unmodified SeP band at 69 kDa (Fig. 2*D*, lane 2). The addition of a 500-fold excess unlabeled SeP completely abolished the appearance of this high-molecular band (Fig. 2*D*, lane 5), suggesting that the formation of this complex is specific. In the case of dithiobis (succinimidyl propionate), which is a thiol-cleavable chemical cross-linker, similar results were obtained under nonreducing conditions (Fig. 2*D*, lanes 6 and 7). Moreover, the 200-kDa complex was only retained in disuccinimidyl suberate–treated cells under reducing conditions (Fig. 2*D*, lanes 8 and 9). After subtracting the molecular mass of SeP (69 kDa) from 200 kDa, the calculated molecular mass of the SeP-binding molecule on Jurkat cells was approximately 130 kDa.

### A 140-kDa ApoER2 variant functions as a receptor for SeP in Jurkat cells

**Figure 3:**
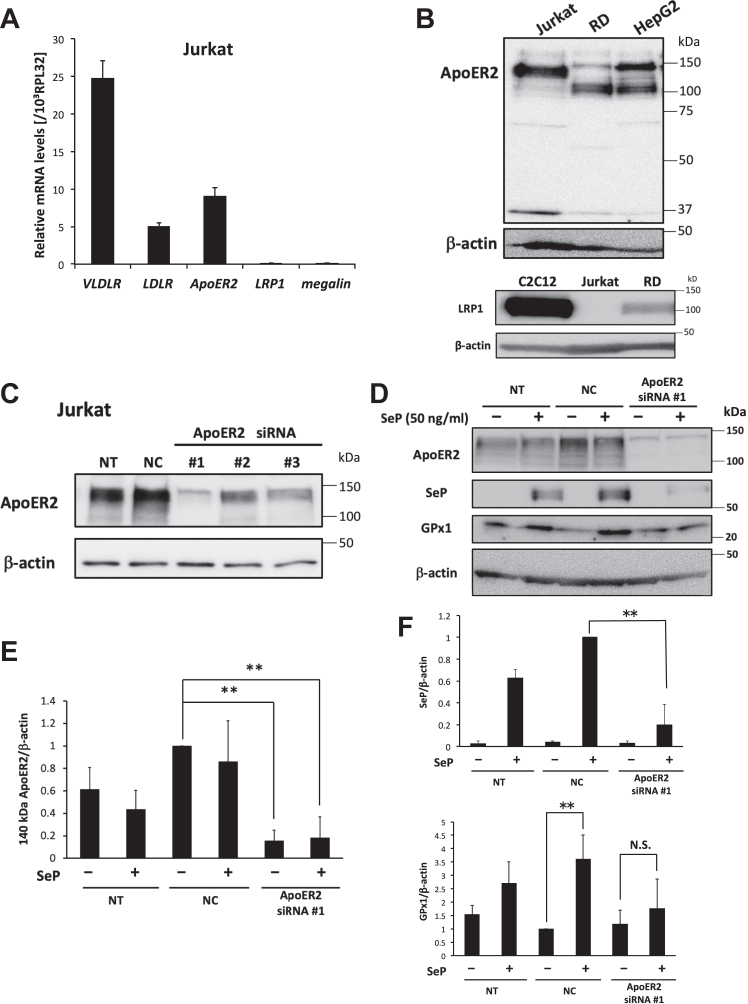
**A 140-kDa ApoER2 variant functions as a receptor for SeP.***A*, relative mRNA levels of lipoprotein receptors in Jurkat cells. Jurkat cells were harvested for RNA isolation and real-time PCR analysis. The expression levels of each lipoprotein receptor were normalized to that of RPL32 mRNA (n = 4, means ± s.d.). *B*, relative protein expression of ApoER2 and LRP1 in each cell line. Whole cell lysates of each cell line were subjected to Western blotting using anti-ApoER2 Ab and anti-LRP1 Ab. *C*, screening of ApoER2 siRNA for Jurkat cells. The cells were treated with each ApoER2 siRNA (#1, #2, and #3) or nonspecific RNA (negative control, NC), cultured with serum- and Se-free media for 72 h. Then, the whole cell lysate was subjected to Western blotting using anti-ApoER2 Ab. *D*–*F*, effect of ApoER2 siRNA on the cellular uptake of human SeP in Jurkat cells. The cells were treated with ApoER2 siRNA #1 or nonspecific RNA (NC), cultured with serum- and Se-free media for 72 h, and then treated with SeP (50 ng/ml) for 24 h. The whole cell lysate was analyzed using Western blotting (n = 3, means ± s.d.). The band intensities of ApoER2 (*E*) and selenoproteins (*F*) were evaluated in each treated condition. ∗∗*p* < 0.01, Tukey ANOVA. ApoER2, apolipoprotein E receptor 2; LRP, lipoprotein receptor–related protein; N.S., not significant; NT, nontransfection; Se, selenium; SeP, selenoprotein P.

We have previously reported that ApoER2 and LRP1 function as SeP receptors in RD cells (19). The expression levels of lipoprotein receptors in human-derived Jurkat and RD cells were determined, and we found ApoER2 expression in both cell types (Figs. 3*A* and S2*B*). Because several splicing variants of ApoER2 are known, we analyzed the molecular weights of the ApoER2 bands. We found a 140-kDa band as a major ApoER2 band using Western blotting in Jurkat cells, while 110-kDa band was observed in RD cells (Fig. 3*B*). In Jurkat cells, the LRP1 protein was not detected in agreement with the result of real-time PCR analysis (Fig. 3*B*). Next, we examined the effects of three kinds of siRNAs for ApoER2. We found that ApoER2 siRNA #1 was the most effective in decreasing the ApoER2 levels (Fig. 3*C*).

We next evaluated the Se-supply activity of SeP in ApoER2-deficient Jurkat cells prepared using ApoER2 siRNA #1 (Fig. 3, *D* and *E*). This siRNA significantly decreased SeP uptake levels (Fig. 3, *D* and *F*). Further, the treatment with ApoER2 siRNA #1 resulted in the elimination of GPx1 elevation induced by SeP addition (Fig. 3, *D* and *F*). These results suggest that a 140-kDa ApoER2 variant functions as a major receptor for SeP uptake in Jurkat cells. In the case of RD cells, ApoER2 siRNA #1 significantly decreased the ApoER2 variants (110 kDa and 150 kDa; Fig. S3*A*) as well as the increase in SeP and GPx1 levels induced by SeP treatment (Fig. S3*B*). This finding suggests that ApoER2 functions as a receptor for SeP uptake in RD cells.

### Role of the O-linked sugar domain in SeP uptake mediated by the 140-kDa ApoER2 variant expressed in Jurkat cells

**Figure 4:**
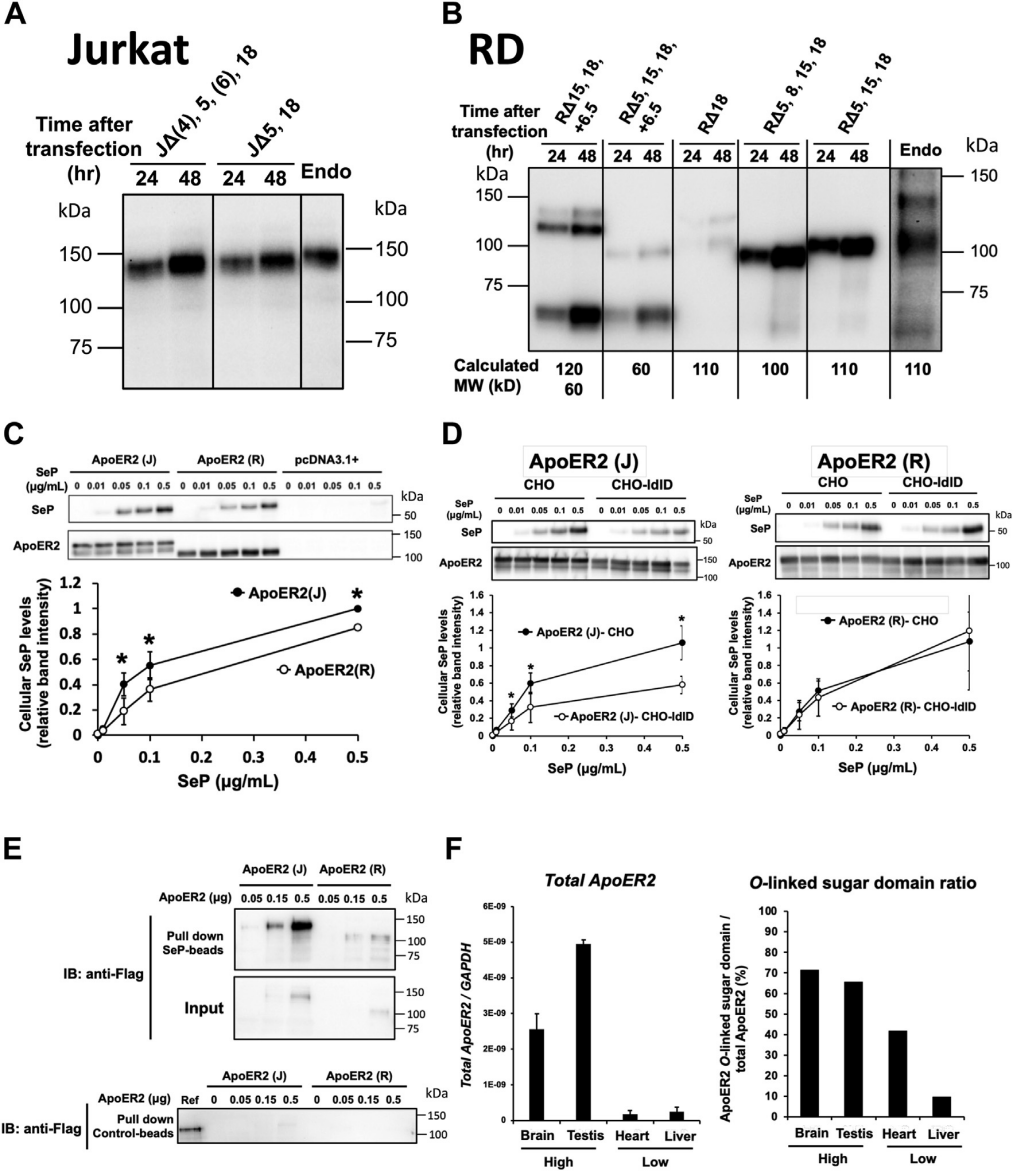
**The role of the *O*-linked sugar domain in the SeP uptake mediated by the 140-kDa ApoER2 variant.***A*, identification of the ApoER2 variants expressed in Jurkat cells. Each variant of ApoER2 was transfected into Jurkat cells 24 h after seeding. After 24 h, whole cell lysates were subjected to Western blotting using anti-ApoER2 Ab. *B*, identification of ApoER2 variants expressed in RD cells. Each variant of ApoER2 was transfected into RD cells. Then, whole cell lysates were subjected to Western blotting. Endo indicates the endogenous expression of ApoER2. The calculated molecular mass of each band is described at the bottom. *C*, comparison of the SeP uptake efficiency of the ApoER2 variants in HEK293T cells. ApoER2(J) (JΔ5,18) and ApoER2(R) (R Δ5,15,18) were transfected into HEK293T cells. Then, purified SeP was added. After 24 h, whole cell lysates were subjected to Western blotting and the band intensities of SeP were evaluated (n = 3, means ± s.d.). ∗*p* < 0.05, Student’s *t* test. *D*, comparison of the SeP uptake efficiency of ApoER2 variants in CHO-ldlD cells. ApoER2(J) and ApoER2(R) were transfected in CHO and CHO-ldlD cells, respectively. Then, the cells were cultured with a Se-deficient medium. After 48 h, purified SeP was added. After 24 h, whole cell lysates were subjected to Western blotting and the band intensities of SeP were evaluated (n = 3, means ± s.d.). ∗*p* < 0.05, Student’s *t* test. *E*, binding assay using soluble ApoER2 recombinant protein. Soluble ApoER2(J) and ApoER2(R) recombinant proteins were added to SeP-conjugated sepharose in 20 mM Tris–HCl (pH 7.5) and incubated for 1 h at 4 °C. After washing, bound ApoER2 was eluted using a sample buffer for Western blotting with anti-Flag Ab. The *upper panel* shows ApoER2 variants bound to SeP-conjugated sepharose, while the *lower panel* indicates bound ApoER2 to control sepharose. *F*, relative expression levels of the ApoER2 gene and the expression ratios of variants in each mouse tissue. RNA isolated from each mouse tissue was subjected to real-time PCR analysis (n = 5, mean ± s.e.m.). *O*-linked sugar domain ratio was calculated from determinants using primers specific to exons 15 and 16, respectively. ApoER2, apolipoprotein E receptor 2; RD, rhabdomyosarcoma; Se, selenium; SeP, selenoprotein P.

Because several splicing variants of ApoER2 have been reported, we analyzed the nucleotide sequence of ApoER2 expressed in Jurkat cells and compared it with that expressed in RD cells. Isolated mRNA forms were evaluated using sequencing analysis to confirm alternative splicing. We isolated two forms of ApoER2 from Jurkat cells and five forms from RD cells (Fig. S4*A*). To identify the major forms of ApoER2 expressed in these cell types, we transfected each isolated ApoER2 variant into the original cells, determined its molecular weight, and compared it with that of endogenous ApoER2. In the case of Jurkat cells, two variants showed similar molecular weights as that of endogenous ApoER2 (Fig. 4*A*). We identified ApoER2 lacking exons 5 and 18 (JΔ5,18) as a major ApoER2 in Jurkat cells because it was the most isolated clone (six of the total eight clones). In the case of RD cells, ApoER2 lacking exons 5, 15, and 18 (R Δ5,15,18) showed the same molecular weight as endogenous ApoER2 (Fig. 4*B*). The difference between JΔ5,18 (described as ApoER2[J]) and R Δ5,15,18 (described as ApoER2[R]) was exon 15 that coded the *O*-linked sugar domain, which altered the proteolytic processing and abundance of ApoER2 in cells. Treatment with glycosidase for N- and O-type sugar chains suggested the glycosylation of ApoER2 in each cell type (Fig. S4*B*). We further investigated the role of the O-linked sugar domain in the cellular uptake of SeP.

ApoER2(J) and ApoER2(R) were transfected into HEK293T cells for evaluation of the cellular uptake of SeP in each ApoER2-expressed cell type. A slight but significant increase in SeP uptake was observed for ApoER2(J) expression compared with ApoER2(R) expression (Fig. 4*C*). To investigate the role of glycosylation in the SeP uptake of each ApoER2 variant, the O-glycosylation–defective CHO-ldlD cells were used (35). We found that in the case of ApoER2(J) expression, the cellular uptake of SeP was significantly decreased in CHO-ldlD cells, while in the case of ApoER2(R) expression, SeP uptake in the CHO-ldlD cells remained unchanged (Fig. 4*D*). Furthermore, soluble ApoER2 recombinant proteins with the Factor Xa cleavage sequence and Flag- and His-tags were prepared using Jurkat and RD cells (Fig. S4*C*). Purified SeP protein was conjugated with N-hydroxysuccinimide-activated sepharose, and soluble ApoER2(J) and ApoER2(R) prepared from each cell line were added to SeP-sepharose. As shown in Figure 4*E*, bound ApoER2 proteins were not detected in control sepharose (lower panel), while in the case of SeP-sepharose, the quantity of bound ApoER2(J) at each concentration was more than that of ApoER2(R) (upper panel). These findings suggest a significant role of the O-linked sugar domain in the binding and cellular uptake of SeP. We evaluated the expression of ApoER2 and O-linked sugar domain in mouse tissues with high and low affinity for SeP and found high expression levels of ApoER2 with the O-linked sugar domain in the brain and testis (Fig. 4*F*).

### Effects of lysosomal inhibitors and role of acidification on the Se-supply activity of SeP in Jurkat and RD cells

**Figure 5:**
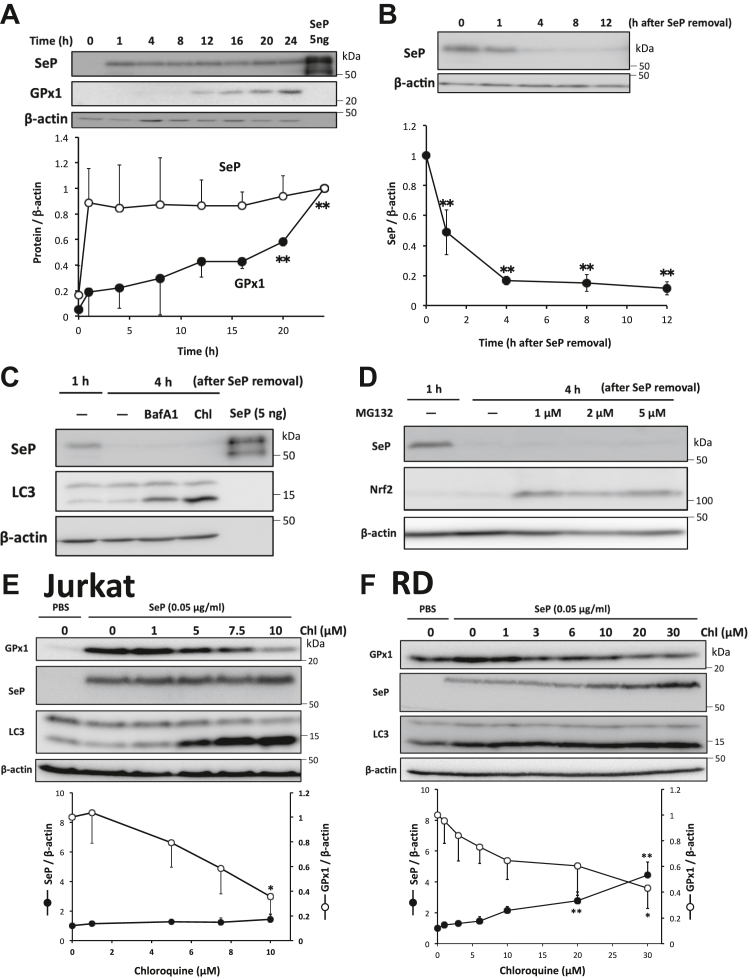
**Effects of lysosomal inhibitor on the Se-supply activity of SeP in Jurkat cells.***A*, time-dependent increase in SeP uptake and GPx1 levels in Jurkat cells. Se-deficient Jurkat cells were cultured with SeP in serum- and Se-free media for the indicated time. Then, whole cell lysates were subjected to Western blotting using anti-SeP Ab and anti-GPx1 Ab (n = 3, means ± s.d.). *B*, Se-deficient Jurkat cells were cultured with SeP in serum- and Se-free media for 24 h. Then, the cells were washed and cultured in serum- and Se-free media for the indicated time. Whole cell lysates were subjected to Western blotting using anti-SeP Ab (n = 3, means ± s.d.). *C* and *D*, Se-deficient Jurkat cells were cultured with SeP in serum- and Se-free media for 24 h. Then, the cells were washed and cultured with 100 nM bafilomycin A1 (BafA1, *C*) and 100 μM chloroquine (Chl, *C*) or the indicated amount of MG132 (d) in serum- and Se-free media for the indicated time. Whole cell lysates were subjected to Western blotting using anti-SeP Ab and anti-LC3 Ab. *E* and *F*, Jurkat and RD cells were cultured with SeP in the presence of Chl for 24 h, and whole cell lysates were subjected to Western blotting (n = 3, means ± s.d.). Band intensities of SeP and GPx1 were evaluated and are shown in the *lower panel*. ∗*p* < 0.05, ∗∗*p* < 0.01, Tukey ANOVA. GPx, glutathione peroxidase; RD, rhabdomyosarcoma; Se, selenium; SeP, selenoprotein P.

Previous studies suggested that the lysosomal degradation of SeP is involved in the Se-supply activity of SeP. A time-dependent study on the Se-supply activity of SeP in Jurkat cells revealed that SeP was incorporated into the cells 1 h after treatment, and GPx1 levels significantly increased 20 h after the addition of SeP (Fig. 5*A*). The incorporated SeP was also detected by immunohistochemistry using anti-SeP Ab (Fig. S5*A*). A significant decrease in the cellular SeP levels was observed from 1 h after the removal of SeP, and cellular SeP almost disappeared 4 h after the removal (Fig. 5*B*). To investigate the involvement of SeP degradation in this process, the additional effects of lysosomal inhibitors, such as bafilomycin A1 (BafA1) and chloroquine, were examined. These inhibitors prevent the acidification of cellular vesicles including lysosomes and inhibit protein degradation in lysosomes. After the treatment of Jurkat cells with SeP for 1 h, cells were further cultured in a serum-free medium without SeP for 3 h in the presence of lysosomal inhibitors. Although the increase in LC3-II formation indicated the inhibition of lysosomal activity, SeP protein levels were not altered in Jurkat cells (Fig. 5*C*). We further investigated the involvement of proteasome degradation using an inhibitor MG132. Nrf2 levels increased on using this inhibitor, which indicated proteasome inhibition, but SeP levels were not increased in Jurkat cells (Fig. 5*D*). This finding suggests that cellular SeP disappeared by the washing process and that the degradation of incorporated SeP was not involved in the Se-supply activity of SeP in Jurkat cells. We also investigated the effects of lysosomal inhibitors on the Se-supply activity of SeP using GPx1 levels as an indicator. We found that the SeP-induced increase in GPx1 levels was significantly inhibited using BafA1 and chloroquine, without any change in cellular SeP levels, suggesting the significant role of acidification on the Se-supply activity of SeP (Fig. S5, *B* and *E*). Sodium selenite treatment increased GPx1 levels in the presence of BafA1 (Fig. S5*B*). In the case of RD cells, BafA1 and chloroquine treatment significantly increased the cellular SeP levels and suppressed the increase in GPx1 levels (Fig. S5, *C* and *F*). This finding suggests the degradation of SeP in the Se-supply process in RD cells. The concentration-dependent study revealed that in the case of Jurkat cells, chloroquine decreased GPx1 levels in a concentration-dependent manner without any change in the incorporated SeP (Fig. 5*E*), while in the case of RD cells, concentration-dependent changes in both GPx1 and SeP levels were observed, namely, chloroquine decreased GPx1 levels and increased SeP levels (Fig. 5*F*). These findings suggest that acidification is necessary for the Se-supply activity of SeP in both cells and that SeP supplies Se to Jurkat cells without undergoing degradation, which completely differs from the way SeP supplies Se to RD cells wherein it undergoes degradation.

### Effects of Sec lyase siRNA on the Se-supply activity of SeP in Jurkat and RD cells

**Figure 6:**
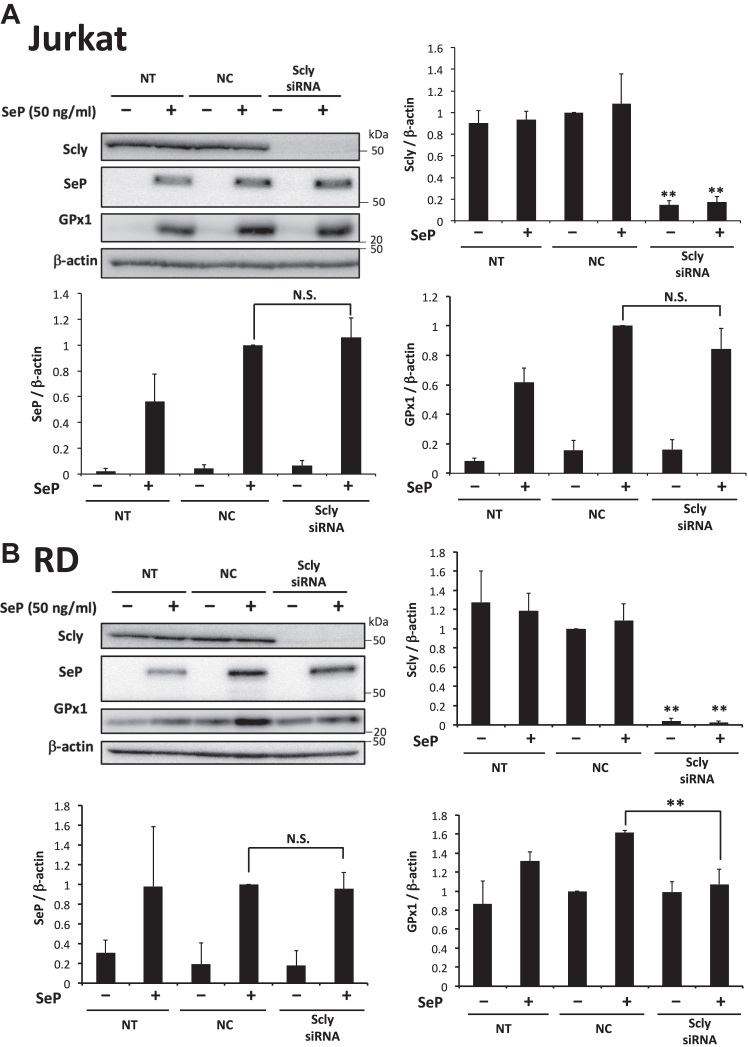
**Selenocysteine lyase–independent Se-supply activity of SeP in Jurkat cells.***A* and *B*, effect of Scly-siRNA on the Se-supply activity of SeP in Jurkat (*A*) and RD (*B*) cells. Each cell type was transfected with Scly-siRNA or nonspecific RNA (negative control, NC) and cultured for 72 h. Then, the cells were treated with SeP (50 ng/ml) for 24 h. The whole cell lysate was subjected to Western blotting (n = 3, means ± s.d.). ∗∗*p* < 0.01, Tukey ANOVA. NT, nontransfection; N.S., not significant; RD, rhabdomyosarcoma; Se, selenium; SeP, selenoprotein P.

The degradation of SeP in lysosomes generates Sec, which is a substrate of Sec lyase, to supply Se for the *de novo* synthesis of selenoproteins. To obtain further insights into the role of lysosomal degradation of SeP, we examined the effects of Sec lyase siRNA on the Se-supply activity of SeP. Sec lyase protein was detected (Fig. S6*A*), and Sec lyase siRNA effectively decreased its protein levels in both RD and Jurkat cells (Fig. S6, *B* and *C*). In the case of Jurkat cells, Sec lyase knockdown did not affect both SeP uptake and the increase in GPx1 levels induced by SeP (Fig. 6*A*), suggesting the nonessential role of Sec lyase in the Se-supply activity of SeP in Jurkat cells. In the case of RD cells, Sec lyase siRNA treatment effectively inhibited the increase in GPx1 levels induced by SeP but not SeP uptake (Fig. 6*B*). These findings suggest that acidification, lysosomal degradation of SeP, and Sec lyase are necessary in RD cells, while in the case of Jurkat cells, acidification is required for the Se-supply activity of SeP, but Sec lyase is not.

### Effects of acidification on the interaction between SeP and ApoER2

**Figure 7:**
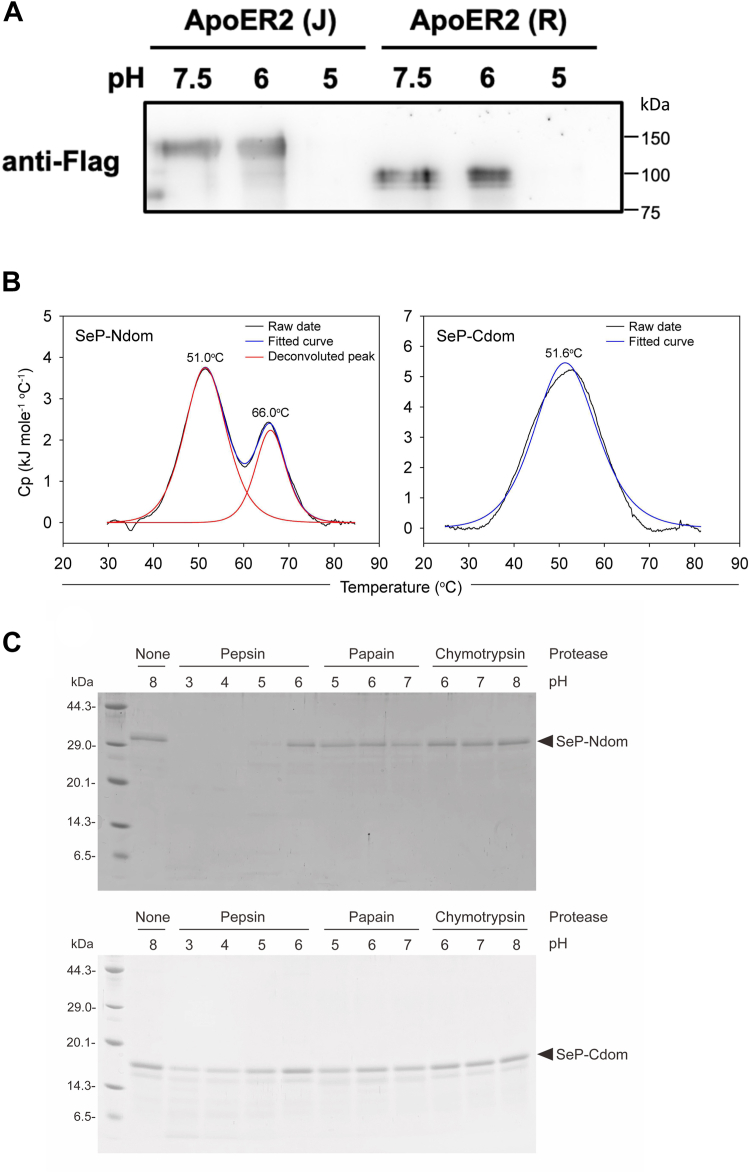
**The effects of pH on the interaction between SeP and ApoER2 and on the susceptibility to proteolytic digestion.***A*, the effects of pH on the interaction between SeP and the ApoER2 variant. Soluble ApoER2(J) and ApoER2(R) recombinant proteins were added to SeP-conjugated sepharose in 20 mM Tris–HCl (pH 7.5) and incubated for 1 h at 4 °C. After washing, the buffer was replaced with each Tris–HCl buffer (pH 5, 6, or 7.5) and the samples were incubated for 1 h at 4 °C. After washing using each buffer, bound ApoER2 was eluted using a sample buffer for Western blotting. *B*, DSC thermograms of the recombinant SeP-Ndom (*left panel*) and SeP-Cdom (*right panel*) were measured at neutral pH of 7.5. *T*_M_ values are indicated. *C*, susceptibility of the recombinant SeP-Ndom (*upper panel*) and SeP-Cdom (*lower panel*) to proteolytic digestion at various pH values. The recombinant proteins were digested with pepsin at pH 3 to 6, papain at pH 5 to 7, or chymotrypsin at pH 6 to 8, and subjected to 15% SDS-PAGE. The gels were stained with CBB. Standard molecular weight markers (*left* lane in each gel) were used. ApoER2, apolipoprotein E receptor 2; DSC, differential scanning calorimetry; SeP, selenoprotein P.

The lumen of the intracellular vesicles possesses low pH that is closely related to its function. We evaluated the effects of pH on the interaction between SeP and ApoER2 variants using the binding assay described above. Soluble ApoER2 recombinant proteins were bound to SeP-conjugated sepharose at pH 7.5. Then, these protein complexes were incubated at pH 5 and 6, which represents the pH of the lumen of lysosomes and early/recycling endosomes, respectively. We found that the binding between SeP and ApoER2 at pH 6 occurred in a variant-independent manner, while these complexes were dissociated under pH 5 (Fig. 7*A*). To understand the effects of pH on the protein structure stability of SeP, additional studies, using the recombinant N-terminal (SeP-Ndom, residues D23-P258) and C-terminal (SeP-Cdom, residues E259-N381) domains of SeP *via* an *Escherichia coli* expression system (Fig. S7*A*), were conducted. We first evaluated the conformational similarity between the recombinant proteins and native SeP, by binding of conformation-specific synthetic binding proteins, termed monobodies (Mbs, Fig. S7*B*) (36). We generated four types of Mbs against SeP-Ndom and 1 Mb for SeP-Cdom, and Mb(SeP_Ndom_S5) and Mb(SeP_Cdom_S3), which showed high affinity to their cognate targets, were used for subsequent analysis (Fig. S7, *C* and *D*). Mb(SeP_Ndom_S5) and Mb(SeP_Cdom_S3) exhibited no detectable binding to SeP-Cdom and SeP-Ndom, respectively, suggesting their high specificity (Fig. S7*E*). Competition-binding experiments using purified SeP as a competitor showed that these Mbs bind native SeP (Fig. S7*F*). These results evidenced the conformational similarity between the recombinant proteins and native SeP, except for glycosylation (Fig. S7, *B*–*F*). We next performed stability analysis of the recombinant proteins. Differential scanning calorimetry (DSC) thermograms of both recombinant proteins showed the *T*_M_ values of 51.0 and 66.0 °C for SeP-Ndom and 51.6 °C for SeP-Cdom, suggesting that both domains have high conformational stability at neutral pH (Fig. 7*B*). To evaluate the effects of pH on the stability of both domains, these recombinants were digested by each protease at indicated pH. The bands of SeP-Ndom and SeP-Cdom were decreased by pepsin treatment from pH 5, suggesting that the destabilization occurs when pH drops below 6.0 (Fig. 7*C*). Taken together, these findings suggest that the binding between SeP and ApoER2 is stably preserved in early/recycling endosomes but not in lysosomes probably due to pH-dependent conformational change of SeP.

## Discussion

Until now, knowledge about the Se transport system *via* SeP was limited. In particular, the association of the diverse ApoER2 variants with this transport system had not been investigated. In the present study using several types of cultured cells, we found that the high-affinity ApoER2 variant with the *O*-linked sugar domain was involved in the efficient transport system for Se in Jurkat cells. In addition, the involvement of a Sec lyase–independent Se transport pathway was clarified. This study revealed a new regulatory mechanism for the efficient transport of Se *via* SeP.

As shown in Figure 1, the efficacy of the SeP-mediated Se transport pathway was high in T lymphoma Jurkat cells and neuroblastoma SH-SY5Y cells, which increased the intracellular GPx levels at lower Se concentrations than selenite, while C2C12 cells and RD cells exhibited low Se utilization *via* SeP. Based on these findings, the efficacy of the use of SeP-derived Se might be evaluated by determining the increase in GPx1 levels in the cells treated with selenite or SeP at a concentration equivalent to 20 nM of Se (0.5 μg/ml of SeP). No significant difference was observed between the four types of cells on treatment with selenite, but in the case of SeP, the order of Se utilization *via* SeP was as follows: Jurkat> SH> RD> C2C12 (Fig. S2). Se utilization *via* SeP was high in cells that required Se for their functions, such as nervous and immune functions, suggesting that these functions contribute to the preferential transport of Se. Among them, C2C12 cells, which are a skeletal muscle model, exhibited the lowest efficiency of Se utilization *via* SeP. Previously, we conducted a Scatchard analysis for C2C12 cells (19). In that case, we could not detect specific binding under 20 ng/ml SeP and the precise Kd value was not estimated (data not shown). In patients with diabetes, increased SeP levels have been demonstrated to induce insulin resistance in the skeletal muscle (16, 18). Moreover, excessive SeP levels induced insulin resistance in C2C12 cells, and it required high concentrations of SeP around the blood concentration of patients with diabetes (10 μg/ml of SeP). The low affinity of the skeletal muscle for SeP is considered to have important implications for suppressing the induction of insulin resistance and the increase in blood glucose levels.

From the binding assay using ^75^Se-labeled SeP, the following parameters regarding the affinity between SeP and its receptor expressed in Jurkat cells were obtained: the dissociation constant Kd value, 0.67 ± 0.33 nM; the number of bindings per cell, 2200 ± 1500. This affinity is at the same level as that of receptors for typical cytokines, such as interleukins, indicating that SeP interacts with Jurkat cells with high affinity (37, 38). No previous study has reported on the Kd value for the binding between SeP and its receptor, and to our knowledge, the present study is the first to report it. We also used the binding assay for C2C12 cells (19). However, these cells exhibited low affinity between SeP and its receptor, and the Kd value could not be determined. The present study demonstrated that the affinity between SeP and its receptor contributes to one of the preferred Se transport mechanisms *via* SeP. In RD cells in which ApoER2 expression is the same as that in Jurkat cells and ApoER2 is used for SeP uptake, the SeP uptake efficiency was low. This finding indicated the different potencies of the ApoER2 variants between these cell types. These observations prompted us to perform cloning of ApoER2 in each of these cell types.

A comparison between the ApoER2 variants predominantly expressed in Jurkat and RD cells revealed that the *O*-linked sugar domain, exon 15, was characteristic of the ApoER2 variant expressed in Jurkat cells. The *O*-linked sugar domain is rich in O-linked glycosylation and Thr and Ser residues (39, 40). The *O*-linked sugar domain contributes to the stability of the ApoER2 levels, depending on the glycosylation level, and to the neural function by stabilizing it (35). We identified minor ApoER2 lacking only exon 18 (RΔ18), which contains the *O*-linked sugar domain, in RD cells. However, its protein expression levels were low (Fig. 4*B*). Glycosylation or loss of *O*-linked sugar domain of ApoER2 protects against specific proteolytic cleavage by metalloprotease and γ-secretase (35). Low protein expression levels of RΔ18 in RD cells might be related to the limited glycosylation in RD cells. Because the glycosylation level differs depending on the cell type, the function of the *O*-linked sugar domain was analyzed using HEK293, CHO-ldlD, and original expressing cells in the present study. ApoER2 containing an *O*-linked sugar domain showed high SeP uptake in each cell type, and the largest difference in SeP uptake was observed in the case of the ApoER2 recombinant proteins expressed in the cell of origin. This finding suggested that the affinity between ApoER2 and SeP was highly dependent on the state of glycosylation. SeP has a region rich in basic amino acids, including a continuous sequence of His residues, which is involved in heparin binding (41). The glycosylation of the *O*-linked sugar domain, probably *via* a negatively charged group, such as a sulfate group, and the positive charge of the heparin-binding site of SeP contribute to the high affinity between the *O*-linked sugar domain and SeP. The Se transport and binding of SeP to cells were almost completely suppressed by heparin addition, suggesting that the sugar chain–mediated interactions play a critical role in these processes (18). However, multiple cell surface molecules undergo glycosylation. Notably, the present study findings cannot deny the possibility that other factors, apart from ApoER2 and its *O*-linked sugar domain, may be involved in the high affinity between SeP and Jurkat cells.

The findings using BafA1 and chloroquine suggested that acidification of cellular vesicles is required for Se transport *via* SeP in both Jurkat and RD cells. Acidification is an important factor that controls the activity of acid hydrolases, such as proteases and lipases, in vesicles and is decisive in lysosomal proteolysis (42, 43). In RD cells, an increase in the intracellular SeP levels was observed following the suppression of acidification, suggesting that Se transport occurs *via* proteolysis in lysosomes. This finding is consistent with the Sec lyase–dependent Se transport shown in studies using the Sec lyase–siRNA, indicating that SeP performs Se transport *via* proteolysis as previously reported (29). However, in the case of Jurkat cells, acidification was necessary, but no increase in SeP levels in the whole cell lyase was observed. These findings suggest that the degradation of SeP protein did not occur, which was in accordance with the Sec lyase–independence of Se transport observed in Jurkat cells. Previously, our research group reported the detection of incorporated SeP using immunostaining and Western blotting (18). These observations implied that SeP taken up into the cells exists in vesicles for a long period. This is strongly supported by the high conformational stability of both the N-terminal and C-terminal domains of SeP. Moreover, glycosylation is known to cause protease resistance due to the steric hindrance, and thus advanced glycosylation of SeP may also contribute to prolonging its intracellular life span (44). In the present study, we further investigated to elucidate the SeP localization. As shown in Fig. S8, we quantified the colocalization of LAM2 (lysosome marker) or Rab11 (Recycling endosome marker) with SeP in both Jurkat and RD cells. These results suggest that dots of Rab11 in Jurkat cells merged slightly but significantly with SeP compared to those of RD cells, while such a difference was not observed in the case of LAMP2 and SeP. In addition, we performed a repeated analysis of the subcellular fractionation and quantified band intensities (Fig. S8). The presence of SeP in recycling endosome fractions was observed; however, its distribution was very low, and a significant difference was not observed. Therefore, at present, the details of whether this difference in the intracellular localization is associated with proteolysis- and Sec lyase–dependence are not yet clear, and the implications of the ApoER2 variants and their cellular localizations need to be clarified in the future. The efficient transport of Se *via* SeP in Jurkat cells might involve not only a high affinity for ApoER2 but also Sec lyase–independent Se transport that is not mediated by proteolysis. Moreover, Sec lyase KO mice did not have a phenotype similar to SeP KO mice; that is, Se transport *via* SeP to the testis was observed in Sec lyase KO mice (33, 34). Based on the present study findings, SeP might transport Se to the testis in a Sec lyase– and proteolysis-independent manner.

In conclusion, the present study revealed the existence of a high-affinity ApoER2 receptor for SeP with an *O*-linked sugar domain and identified a novel Se transport mechanism *via* SeP that is Sec lyase–independent. Further research is needed to identify the molecules that differentiate intracellular vesicles and the possibility of Se being released from SeP in the Sec lyase–independent Se transport pathway.

## Experimental procedures

### Chemicals

Diisopropyl fluorophosphates, BafA1, and chloroquine diphosphate were purchased from Wako Pure Chemical. RPMI 1640 medium, selenocystine, selenomethionine, recombinant human insulin, human transferrin, and 3,3′,5,5′-tetramethylbenzidine were purchased from Merck. Dulbecco’s modified Eagle’s medium (DMEM), penicillin and streptomycin, and horse serum (HS) were obtained from Invitrogen (Thermo Fisher Scientific). ^75^Se as selenite was purchased from the University of Missouri Research Reactor Facility. Vitamin E isoform, α-tocopherol, was kindly provided by Eisai. Human SeP was purified from human plasma (9). Human plasma was mixed with PEG, and then proteins in the supernatant were separated by heparin-Sepharose CL-6B column, Q-Sepharose Fast Flow, and Ni-NTA-agarose, respectively. The buffer of purified SeP was changed by using a PD-10 gel filtration column equilibrated with the desired buffer. Human frozen plasma was kindly provided from the Japanese Red Cross Kinki Block Blood Center (No. 25J0012). All other chemicals used were of the highest quality commercially available.

### Cell culture

All cell lines used were obtained from the American Type Culture Collection. Human T-cell lymphoma Jurkat cells were maintained in RPMI 1640 medium containing 10% heat-inactivated fetal bovine serum (FBS) and antibiotics. Human RD cells, mouse myoblast C2C12 cells, and human hepatoma HepG2 cells were routinely maintained in DMEM containing 10% heat-inactivated FBS and antibiotics. In the case of human neuroblastoma SH-SY5Y cells, DMEM/F12 containing 10% heat-inactivated FBS and antibiotics were used. All cell lines were cultured at 37 °C under an atmosphere of 95% air and 5% CO_2_. To induce myogenic differentiation, the C2C12 cells were subsequently maintained in DMEM containing 0.5% HS and antibiotics.

### Western blotting

To obtain whole cell lysates, treated cells were suspended in lysis buffer (50 mM Tris–HCl pH 7.5, 150 mM NaCl, 1% NP40, 0.1% SDS, 1% sodium deoxycholic acid with a cocktail of protease inhibitor (Nacalai Tesque) and phosphatase inhibitor (PhosSTOP, Roche)) at 4 °C for 30 min. Nuclei and unlysed cellular debris were removed by centrifugation at 15,000×*g* for 5 min. The protein concentration was determined by using a bicinchoninic acid protein assay kit (Pierce Biotechnology) with bovine serum albumin (BSA) as the standard. The protein samples were separated by SDS-PAGE, and then the separated proteins were transferred to an Immobilon-P Transfer Membrane (Millipore). The membranes were blocked in 5% skimmed milk powder (Snow Brand Milk Products) dissolved in Tris-buffered saline (pH 7.4) containing 0.1% Tween 20 (TBS-T) and incubated with appropriate antibodies. Rat anti-hSeP mAbs (Clone BD1) (45) and rat anti-TrxR1 mAb KB12 (46) were used at 1 μg/ml for Western blotting. The following antibodies were used for Western blotting: rabbit anti-GPx1 pAb (for mouse samples, ab22604; Abcam), mouse anti-GPx1 mAb (for human samples, clone GPX-347, M015-3; Medical Biological Laboratories), mouse anti-β actin mAb (clone AC-15; Merck), rabbit anti-ApoER2 mAb (for C-terminal, clone EPR3326, ab108208, Abcam), rabbit anti-LRP1 mAb (clone EPR3724, ab92544, Abcam), mouse anti-LC3 mAb (Clone LC3.No.6, CTB-LC3-1-50, CosmoBio), rabbit anti-Nrf2 pAb (H-300, sc-13032, Santa Cruz Biotechnology), and rabbit anti-Sec lyase pAb (ab169474, Abcam). Treated membrane was incubated with HRP-conjugated secondary antibodies for at least 1 h, and the immunoreactivity was visualized with Immobilon Western (Millipore) and an LAS-4000 luminescence imager (Fujifilm). The relative densities were determined with the MultiGauge software (Fujifilm; https://www.ualberta.ca/biological-sciences/media-library/mbsu/fla-5000/mulitgauge20.pdf). For silver staining, the separated proteins were stained with the Dodeca Silver Stain Kit (Bio-Rad Laboratories).

### SeP uptake and Se-supply assay

SeP uptake and Se-supply activities were examined by the Western blot analysis of SeP and selenoprotein levels such as GPx1 and TrxR1 in the whole lysate of treated cells, respectively. In the case of Jurkat and SH-SY5Y cells, cells were cultured serum- and Se-free medium (basal medium containing 5 μg/ml insulin, 5 μg/ml transferrin with 92 nM FeCl_3_, 2.5 mg/ml BSA, and 2 μM α-tocopherol) for 72 h, which resulted in the undetectable levels of SeP and GPx1. In the case of C2C12 cells, differentiation to myocytes was induced by DMEM containing 0.5% HS for 72 h, which resulted in undetectable levels of SeP and GPx1. In the case of RD cells, since SeP and GPx1 levels were low, RD cells cultured with serum medium were used.

These cells were treated with the indicated concentration of purified human SeP protein or sodium selenite for 24 h, and then cells were washed and whole cell lysates were subjected to Western blotting.

### Preparation of ^75^Se-labeled SeP

The ^75^Se-labeled SeP was prepared by HepG2 cells, which expressed SeP endogenously. For the preparation of ^75^Se-labeled SeP, confluent cells in a 175-cm^2^ flask (Nunc, Thermo Fisher Scientific) were rinsed twice with PBS and 23 ml of serum-free DMEM was added. Two days later, the cells were washed twice with serum-free DMEM and then cultured with 23 ml of serum-free DMEM containing 3 μCi/ml ^75^Se as selenite for 3 days. A labeled protein in this preparation was chromatographed on a Ni-NTA agarose column. The medium was collected, and imidazole was added at a final concentration of 2 mM. The fractions were applied to a column (1 ml) of Ni-NTA agarose equilibrated with 20 mM Tris–HCl, pH 8.0, containing 2 mM imidazole, at a flow rate of 30 ml/h. After washing with 20 mM Tris–HCl, pH 8.0, containing 20 mM imidazole and 1 M NaCl, the bound proteins were eluted with 20 mM Tris–HCl, pH 8.0, containing 250 mM imidazole and 1 M NaCl. For further study, imidazole and NaCl were removed by passage through a PD-10 gel filtration column equilibrated with PBS. 0.1 % crystallized BSA was added to the partially purified ^75^Se-labeled SeP before storage at −80 °C. The concentration of SeP was determined by a sandwich ELISA (47). Prepared ^75^Se-labeled SeP was subjected to SDS-PAGE. ^75^Se-labeled proteins were visualized on SDS-PAGE dried gels with a FUJIFILM BAS-2500 (GE Healthcare). For immunoprecipitation analysis, immobilized six mAbs against human SeP (Clone: BD1, BD3, BF2, AE2, AH5, and AA3) and anti-TrxR1 mAb KB12 were used.

### SeP-binding assay

The binding of ^75^Se-labeled human SeP to the cells was measured according to the methods described with a slight modification (48). To remove endogenous SeP prior to the binding assay, Jurkat cells were incubated for 2 h at 37 °C in the serum- and Se-free medium described above. The cells (1.4 × 10^7^) were further incubated with a serial dilution of ^75^Se-labeled SeP for 1 h at 4 °C in a total volume of 180 μl of RPMI 1640 containing 0.1% BSA, 0.1% NaN_3_, and 50 mM Hepes (pH 7.4). Then, 50 μl of the cell suspension was centrifuged through a 250-μl layer of a mixture of 20% olive oil/80% di-n-butyl phthalate (Nacalai) for 5 min at 10,000*g*. The tips of the tubes containing the cell pellets were cut off, and the radioactivity was measured in a γ–counter (Wallac Wizard 1470, PerkinElmer). Nonspecific binding was determined in the presence of a 500-fold excess of unlabeled SeP.

### Cross-linking study

^75^Se-labeled SeP was chemically cross-linked with the cells as described previously with a minor modification (49). Cells (2.7 × 10^7^) were incubated for 1 h at 4 °C with 50 ng/ml ^75^Se-labeled SeP and a 500-fold excess of unlabeled proteins in a total volume of 300 μl of RPMI 1640 containing 0.1% BSA, 0.1% NaN_3_, and 50 mM Hepes (pH 7.4). Hundred microliters of the incubation mixture was centrifuged at 1500*g* for 5 min. The pellets were resuspended in 200 μl of PBS and centrifuged at 10,000*g* for 5 min through 800 μl layers of 10% sucrose in PBS. The pellets were suspended in 400 μl of PBS containing 1 mM magnesium chloride and 0.02% NaN_3_ (pH 8.3, adjusted by 0.1 N NaOH), and disuccinimidyl suberate or dithiobis (succinimidyl propionate) (Pierce) in dimethyl sulfoxide was added to a final concentration of 1 mM. After incubation at 4 °C for 30 min, the reaction was quenched by the addition of 800 μl of 10 mM Tris, pH 7.4, containing 1 mM EDTA and 0.14 M NaCl. After 5 min, cells were pelleted by centrifugation at 10,000*g* for 5 min. The pellets were solubilized with 100 μl of tris-buffered saline containing 0.5% NP-40, a cocktail of protease inhibitor, and 2 mM diisopropyl fluorophosphate at 4 °C for 20 min. After centrifugation at 10,000*g* for 10 min, the supernatant was subjected to SDS-PAGE in slab gels (7.5%) under nonreducing or reducing conditions. ^75^Se-labeled proteins were visualized on SDS-PAGE dried gels with a FUJIFILM BAS-2500 apparatus. In a large-scale experiment, 1.0 × 10^8^ cells were used.

### Real-time PCR analysis

Total RNA was extracted from each cultured cell using Tripure isolation reagent (Roche) and then reverse-transcribed using a Prime-Script RT reagent kit (TaKaRa Bio). Quantitative real-time PCR was performed using Power SYBR Green PCR Master Mix (Invitrogen) with the 7900HT Fast Real-Time PCR System (Applied Biosystems) according to the manufacturer’s instructions. The housekeeping gene of ribosomal protein L32 (RPL32) was used as an endogenous control. The primers for amplification were as follows: human RPL32, 5′-CCC CTT GTG AAG CCC AAG A-3′ (forward), 5′-TGA CTG GTG CCG GAT GAAC-3′ (reverse); human ApoER2, 5′-GTT GCC ACC AAT CGC ATCT-3′ (forward), 5′-TCG GGT CAC TGG CCT TGT-3′ (reverse); human LRP1, 5′-CCA TTT ACT CAG CCC GTT ACG-3′ (forward), 5′-GTG TGT TTG TTC GCC AGT CAGT-3′ (reverse); human megalin, 5′-TGA ACG TCA AGA TTG CTC ACAA-3′ (forward), 5′-GAC GTG GTC GCA CCT GTA TTC-3′ (reverse); human VLDLR, 5′-TGC CAG CAC CAC AGA TTA ATG-3′ (forward), 5′-TTG TAC CCA CTG GGA CAG GAA-3′ (reverse); human LDLR, 5′-AAG CCA TTC ACT TCC CCA ATC-3′ (forward), 5′-GCC TCA CCG TGC ATG TTT TA-3′ (reverse).

### Transfection of siRNA

The human ApoER2 siRNAs were designed and manufactured by Thermo Fisher Scientific, according to the current guidelines for effective knockdown by this method. The target sequences for human ApoER2 siRNA (Thermo Fisher Scientific), catalog number HSS11751 (#1), HSS11752 (#2), and HSS187874 (#3), were used. The siRNA for human Sec lyase (HSS122331) was purchased from Thermo Fisher Scientific. The siRNAs were transfected into Jurkat cells by electroporation (Neon, Thermo Fisher Scientific). After transfection, treated Jurkat cells were cultured with a Se-deficient medium for 72 h and used for SeP uptake and Se-supply assay. In the case of RD cells, the siRNA was transfected by Lipofectamine RNAi MAX (Thermo Fisher Scientific). After transfection, treated RD cells were cultured for 72 h and used for further experiments.

### Cloning of ApoER2 in Jurkat and RD cells and transfection

Total RNA was extracted from Jurkat and RD cells using Isogen II (Nippon Gene) and then reverse-transcribed using a Prime-Script RT reagent kit (TaKaRa Bio). Based on the sequence of human LRP8 Isoform1 (NCBI), the human ApoER2 gene was first amplified *via* PCR using primers outside of CDS, Nested PCR: 5′-GTCACCGAACCTGCTTGAAATG-3′ (forward), 5′-AAATCACACACACACATACACTCAC-3′ (reverse), and then PCR product was further amplified *via* PCR using primers of exon2, 5′-TGGAATTCTGCAGATAAGGATTGCGAAAAGGACCAATTC-3′ (forward), exon19, 5′-GCCACTGTGCTGGATTCAGGGTAGTCCATCATCTTCAAGGCTTAATGC-3′ (reverse). Cloned ApoER2 genes were inserted into pcDNA3.1 (Thermo Fisher Scientific) using an In-Fusion HD Cloning Kit. After the sequence analysis, exon1 was inserted by following primers: exon1 5′-AGCTTAAGTTTAAACGCTAGCC-3′ (forward), exon4 5′-ACCCACCAGCCACAAGTGTG-3′ (reverse). Finally, the ApoER2 genes were amplified by following primers: insert F 5′-GTTTAAACTTAAGCTATGGGCCTCCCCGAGCC-3′ (forward), insert R 5′-TTGTGGCTGGTGGGTCCACAGCTCA-3′ (reverse). Full sequence of ApoER2 mRNA was confirmed by Sanger sequencing (FASMAC). Resulting plasmids were transfected to RD, HEK293T, CHO, and CHO-ldlD cells using PEI, as described previously (50). In the case of Jurkat cells, electroporation was used.

### Preparation of soluble ApoER2 recombinant proteins

ApoER2(J) and ApoER2(R) gene were amplified by following primers: ApoER2(J)_cut501 5′-ACCATCACATCGAAGGTCGTTATGCAAATG-3′ (forward), ApoER2(J)_cut.301 5′-TCGTCATCGTCCTTGTAATCGTGCTGGGAG-3′ (reverse); ApoER2 (R)_cut.501 5′-ACCATCACATCGAAGGTCGTGATGCAAATG-3′ (forward), ApoER2 (R)_cut.301 5′-TCGTCATCGTCCTTGTAATCTCGGTAGCAC-3′ (reverse). Then, the cleavage sequence of Factor Xa, His-tag, and Flag tag was inserted by using the following primers: ApoER2 (J), (R)_cut.502 5′-CTTCCAATCGCATCACCATCACCATCACAT-3′ (forward), ApoER2 (J), (R)_cut.302 5′-TACAGGTTCTCCTTGTCGTCATCGT-3′ (reverse). Each plasmid of ApoER2(J)_cut and ApoER2(R)_cut was transfected to Jurkat or RD cells, respectively, and the conditioned medium was collected 48h after transfection. Each ApoER2 recombinant protein of soluble form was purified by Ni-NTA agarose, and the buffer of partially purified soluble ApoER2 was changed to PBS by using dialysis and used for further experiments.

### Binding assay between SeP and ApoER2

Purified SeP protein was covalently conjugated to N-hydroxysuccinimide-activated Sepharose (Merck) in 0.2 M NaHCO_3_ containing 0.5 M NaCl (pH 8.3), and then the remaining unreacted residues were treated with blocking buffer (0.2 M NaHCO_3_ containing 0.5 M ethanolamine and 0.5 M NaCl, pH8.3). Soluble ApoER2 recombinant proteins were added to SeP-conjugated Sepharose in 20 mM Tris–HCl containing 2 mM CaCl_2_ (pH 7.5) and rotated at 4 °C for 1 h. After washing, bound ApoER2 was eluted by sample buffer for Western blotting. To evaluate the effects of pH on the interaction between SeP and ApoER2, after the reaction at pH 7.5 for 1 h, Sepharose was washed by Tris–HCl buffer (pH7.5), and then the buffer was replaced with each Tris–HCl buffer with pH7.5 or pH6 or pH5 and rotated at 4 °C for 1 h. After washing by each buffer, bound ApoER2 was eluted by sample buffer.

### Immunohistochemistry

Treated cultured cells were fixed in PBS containing 4% paraformaldehyde for 1 h and then immunostained. Incorporated human SeP protein in the cells was visualized by using rat anti-human SeP mAb BD1 (5 μg/ml) and Alexa 488–conjugated anti-rat IgG (Molecular Probes). Hoechst 33258 dye (Dojindo) was used to stain cell nuclei. The dilution of each Ab was determined according to the instruction. Specimens were observed using a laser-scanning confocal fluorescence microscope (LSM 710 ConfoCor 3; Carl Zeiss and FV1000; Olympus) equipped with Zeiss Efficient Navigation 2009 software (https://www.zeiss.com/microscopy/en/products/software/zeiss-zen.html). For pixel distribution analysis, the stained cells were observed by laser-scanning confocal fluorescence microscope FV1000 and analyzed by Fluoview software (https://www.olympus-lifescience.com/ja/support/downloads/).

### Preparation of the recombinant N-terminal and C-terminal domains of SeP from *Escherichia coli*

The N-terminal domain of SeP (SeP-Ndom, residues D23-P258), in which the putative active site Sec in a position 59 was mutated to Cys, was prepared as a fusion protein C-terminal to a NusA-tag and a TEV cleavage site or another fusion protein C-terminal to a NusA-tag and a TEV cleavage site and N-terminal to a biotin-acceptor tag (Avi-tag) using pCold I vector (TaKaRa Bio). The C-terminal domain of SeP (SeP-Cdom, residues E259-N381), in which nine Sec residues in positions 300, 318, 330, 345, 352, 367, 369, 376, and 378 were mutated to Cys, was prepared as a fusion protein C-terminal to His6, a NusA-tag, and a TEV cleavage site or another fusion protein C-terminal to His6, a NusA-tag, and a TEV cleavage site and N-terminal to an Avi-tag using pCold I vector. Nonbiotinylated proteins without an Avi-tag were produced in Origami2(DE3) cells (Novagen). Biotinylated proteins with an Avi-tag were produced in Origami2(DE3) cells containing the pBirAcm plasmid (Avidity) in the presence of 50 μM D-biotin for *in vivo* biotinylation. These derivatives were purified using a 5-mL HisTrap HP column (Cytiva), followed by removal of His- and NusA-tags using TEV protease and further purification with a Superdex200 size-exclusion column (Cytiva).

### Mb generation

The biotinylated SeP-Ndom and SeP-Cdom were used as targets for phage display selection from the “side” Mb library as previously described (51, 52). Four rounds of selection were performed at target concentrations of 100 nM for rounds 1 to 2 and 50 nM for rounds 3 to 4, respectively. After gene shuffling among phage clones within each enriched population and transfer of the resulting gene pool to a yeast-surface display vector, we performed library sorting using the target concentrations of 250 nM and 25 nM for the first and second round sorting, respectively, as described previously (36, 53). Eight clones exhibiting moderate to strong binding affinity for each domain were selected for sequencing, yielding four unique clones for SeP-Ndom and one unique clone for SeP-Cdom. Affinity of generated Mbs was determined using yeast-surface display as described previously (51).

### Competition binding assay

Competition binding assay was performed using yeast surface display as described above, except that Mbs displayed on yeast cells were pre-incubated with excess native SeP competitor (2.67 μM) for 30 min on ice, and then the mixture was transferred to the wells where Mb binding to the biotinylated recombinant SeP-Ndom or SeP-Cdom took place. For this assay, the concentrations of biotinylated proteins used were both 300 nM.

### DSC measurements

For the DSC measurements, the nonbiotinylated proteins were dialyzed against appropriate buffers. SeP-Ndom (1.0 mg/ml) was dialyzed against 20 mM Tris–HCl (pH 7.5) containing 0.5 M NaCl, 0.1 mM DTT, and 10% (v/v) glycerol. SeP-Cdom (1.0 mg/ml) was dialyzed against 20 mM Tris–HCl (pH 7.5) containing 0.5 M NaCl, 1.0 mM EDTA, and 10% (v/v) glycerol. DSC measurements were conducted using the MicroCal VP-Capillary DSC system (Malvern Panalytical) up to 85 °C at a scan rate of 1.0 °C/min. Data collected were analyzed using the Origin 7 program (OriginLab), and baseline-subtracted data were fitted to a non-two-state fitting model to obtain apparent *T*_M_ values.

### Proteolytic susceptibility

The nonbiotinylated SeP-Ndom and SeP-Cdom were incubated at 37 °C for 4 h with pepsin (Sigma-Aldrich) at pH 3 to 6, papain (Nacalai Tesque) at pH 5 to 7, or chymotrypsin (Nacalai Tesque) at pH 6 to 8. Protease/substrate ratios used were 1:500 (w/w) for pepsin, 1:1000 (w/w) for papain, and 1:5000 (w/w) for chymotrypsin. These protease/substrate ratios were chosen as each protease almost fully degrades casein when reacted at the selected ratio and 37 °C for 4 h. The proteolytic reaction was quenched after 4 h by addition of 2× SDS sample buffer to a final concentration of 1× and boiling, and the digestion products were analyzed *via* SDS-PAGE. The buffers used were as follows: pH 3 to 6, 0.1 M sodium citrate buffer; pH 5 to 7, 0.1 M sodium phosphate buffer; pH 6 to 8, 0.1 M Tris–HCl buffer.

### Subcellular fractionation by discontinuous sucrose density gradient centrifugation

The sucrose density gradient fractionation was performed with slight modifications (54). Jurkat cells or RD cells were seeded on a 10 cm dish (2.5 × 10^6^ cells/dish respectively) and incubated for 24 h. Then, purified SeP (0.5 μg/ml) was added to the medium and further incubated for 6 h. The cells were harvested and washed with ice-cold PBS; then the cells were suspended in 5 ml of ice-cold 10 mM Tris–HCl (pH7.4) and osmotically swelled for 1 min. After the centrifugation (200*g* for 2 min at 4 °C), the cells were resuspended in the 10 mM Tris–HCl (pH7.4) buffer containing 1 mM EGTA, 0.5 mM EDTA, and 0.25 M sucrose and then homogenized with five strokes of a Dounce homogenizer. After that, every experimental step was performed on ice or at 4 °C. The homogenized product was centrifuged at 1000*g* for 10 min at 4 °C, and the supernatant was collected (the precipitate is nuclear). Then the supernatant was centrifuged again (8000*g* for 20 min at 4 °C), and an aliquot (1 ml) of supernatant was mixed with an equal amount of 100 mM sodium carbonate (pH 11) and incubated for 5 min on ice. The mixture was sonicated by ultrasonic disintegrator (Microtec) and transferred to a polycarbonate ultracentrifuge tube. Equal amount (2 ml) of 80% (w/v) sucrose in 10 mM Tris–HCl (pH7.4), 1 mM EGTA, 0.5 mM EDTA was added and adjusted to 40% sucrose and 4 ml as final volume. Next, the mixture was overlaid with 4 ml of 35% sucrose in 10 mM Tris–HCl (pH 7.4), 1 mM EGTA, 0.5 mM EDTA and 4 ml of 5% sucrose in 10 mM Tris–HCl (pH 7.4), 1 mM EGTA, 0.5 mM EDTA subsequently. Then, density gradient centrifugation was performed by Himac CP900WX (Hitachi) at 180,000*g* overnight at 4 °C. After that, aliquot (1 ml) of gradient fractions was collected from the top of the tube (12 fractions were obtained) and stored at −20 °C before use.

### Statistical analysis

Data are shown as the mean ± SD. Statistical analyses were performed using Excel software (https://microsoft-excel.en.softonic.com/mac) and IBM SPSS (https://www.ibm.com/jp-ja/products/spss-statistics). Differences between the two groups were assessed using a two-tailed unpaired Student's *t* test. Data involving more than two groups were assessed by ANOVA as described in the figure legends. *p* < 0.05 was considered to indicate significant differences.

## Data availability

The datasets were not generated during this study.

## Supporting information

This article contains supporting information.

## Conflict of interest

The authors declare that they have no conflict of interest with the contents of this article.
