# Supplementary material for: An efficient selenium transport pathway of selenoprotein P utilizing a high-affinity ApoER2 receptor variant and being independent of selenocysteine lyase
Source: J Biol Chem. 2023 Jul 3;299(8):105009. doi: 10.1016/j.jbc.2023.105009 (PMC10407282; doi:10.1016/j.jbc.2023.105009)
Supplement: Supplementary Figures [file mmc1.pdf]

Supplementary figures

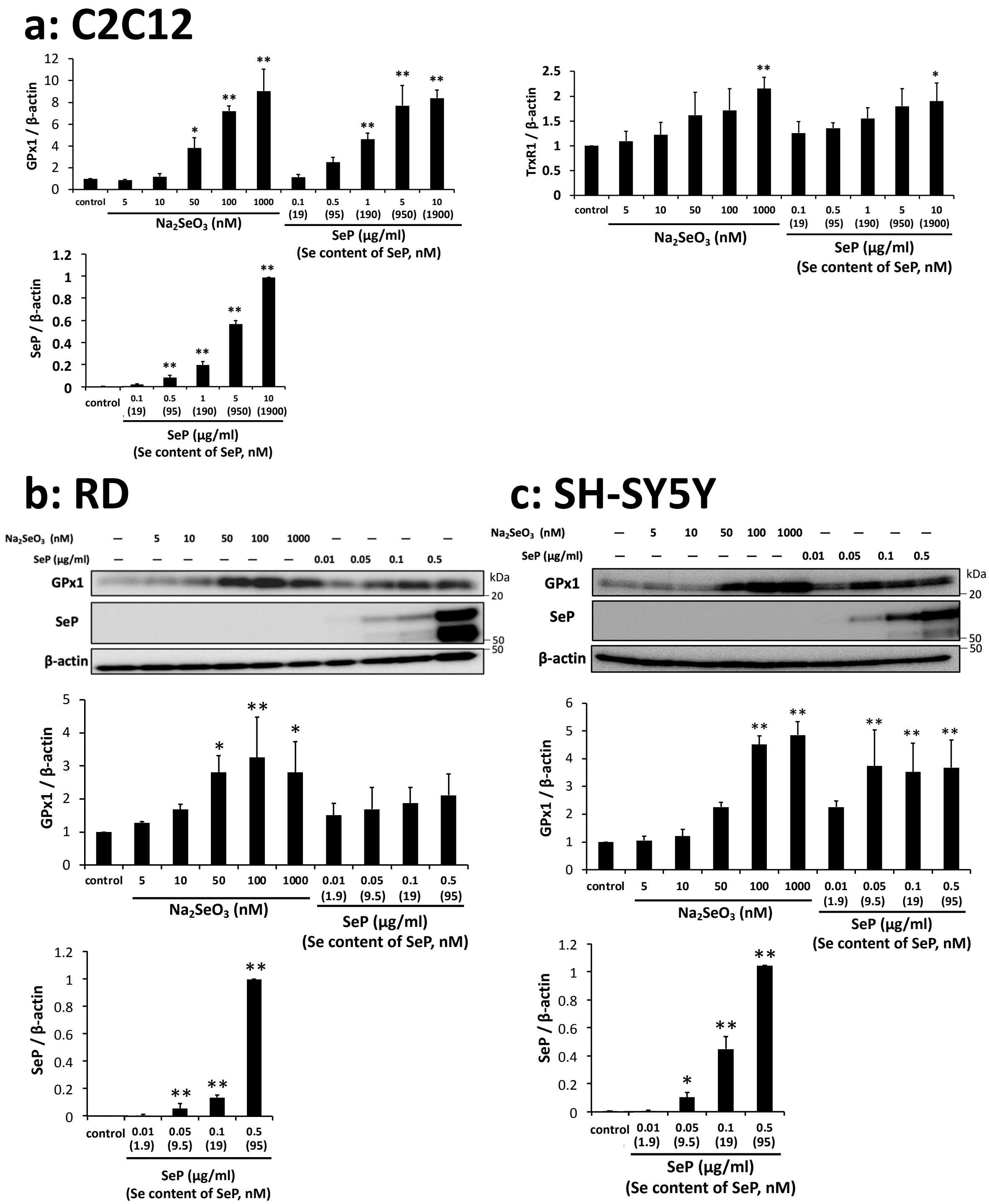

Supplementary Fig S1, Mizuno et al.,

Supplementary Fig. S1 | Different Se-supply activities of SeP depending on the cell type. **a-c** Cellular uptake and Se-supply activity of SeP in mouse C2C12 myocytes (**a**), human rhabdomyosarcoma RD cells (**b**), and human neuroblastoma SH-SY5Y cells (**c**). Each cell type was treated with the indicated concentration of sodium selenite (Na_2SeO_3) and purified human SeP protein for 24 h. Then, whole cell lysates were analyzed using western blotting with anti-GPx1 Ab, anti-TrxR1 Ab KB12, and anti-SeP Ab BD1. GPx1, TrxR1, and SeP levels in the whole cell lysates were determined using western blotting (n = 3, means ± s.d.). The band intensity of SeP was only evaluated in SeP-treated cells. $**P < 0.01$, $*P < 0.05$, vs. control, Tukey ANOVA.

a

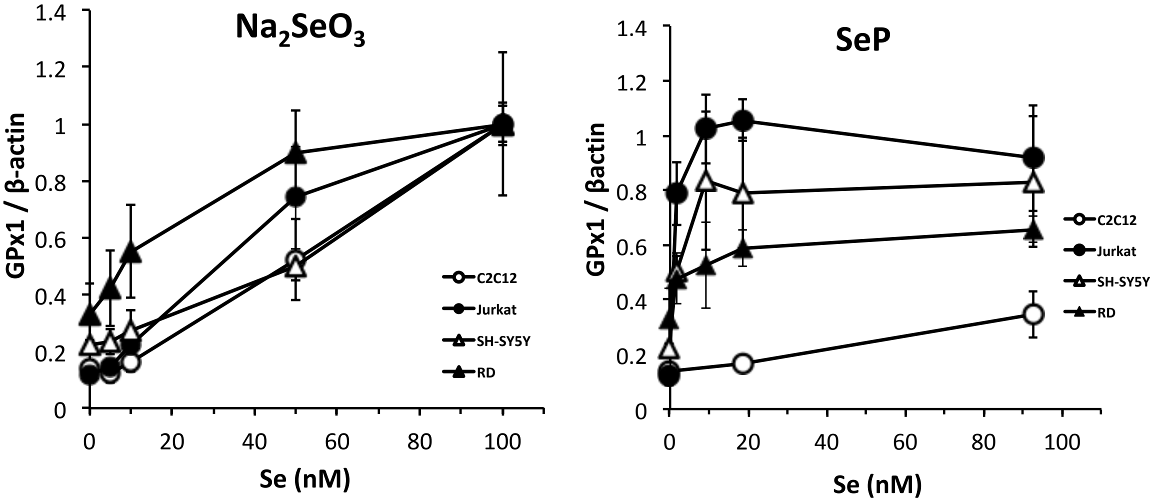

b: RD

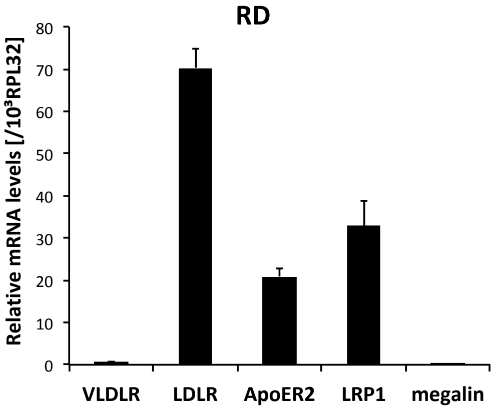

Supplementary Fig S2, Mizuno et al.,

Supplementary Fig. S2 | Cell type-dependent Se-supply activity of SeP. **a** Comparison of the Se-supply activity of SeP with that of sodium selenite in each cell type. Relative GPx1 levels in Na_2SeO_3- or SeP-treated cells were plotted against selenium concentration of each condition (n = 3, means ± s.d.). **b** Relative mRNA levels of lipoprotein receptors in RD cells. The cells were harvested for RNA isolation and real-time PCR analysis. The expression level of each lipoprotein receptor was normalized to that of RPL32 mRNA (n = 4, means ± s.d.).

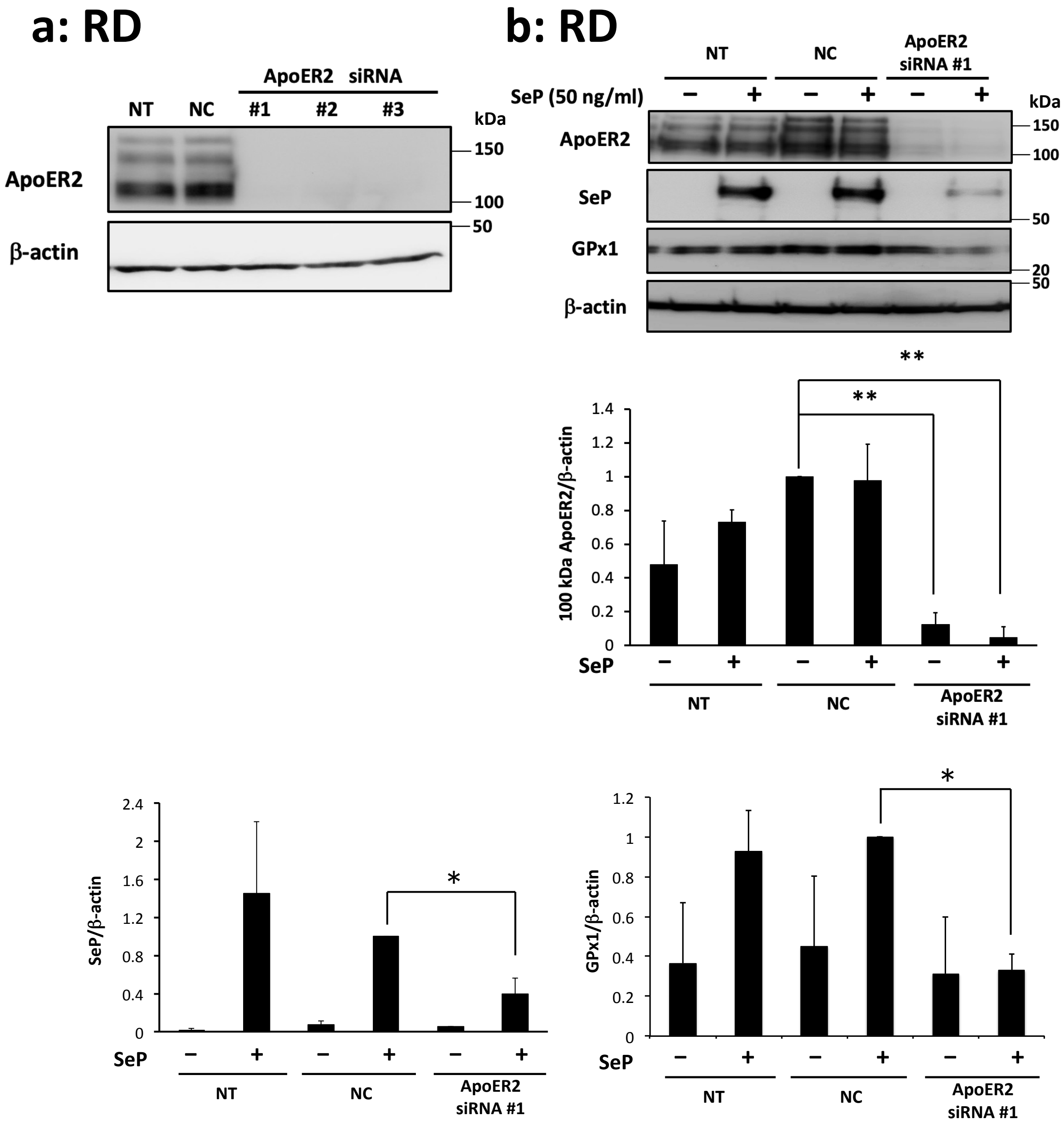

Supplementary Fig S3, Mizuno et al.,

Supplementary Fig. S3 | Effect of ApoER2-siRNA on ApoER2 levels and the cellular uptake of human SeP in RD cells. **a** The cells were treated with each ApoER2-siRNA or non-specific RNA (NC) and cultured in a serum medium for 72 h. The whole cell lysate was analyzed using western blotting. **b** RD cells were treated with ApoER2-siRNA#1 and then with SeP (50 ng/mL) for 24 h.

The whole cell lysate was analyzed using western blotting (n = 3, means ± s.d.). $^{**}P < 0.01$, $^{*}P < 0.05$, Tukey ANOVA. NT: non-transfection.

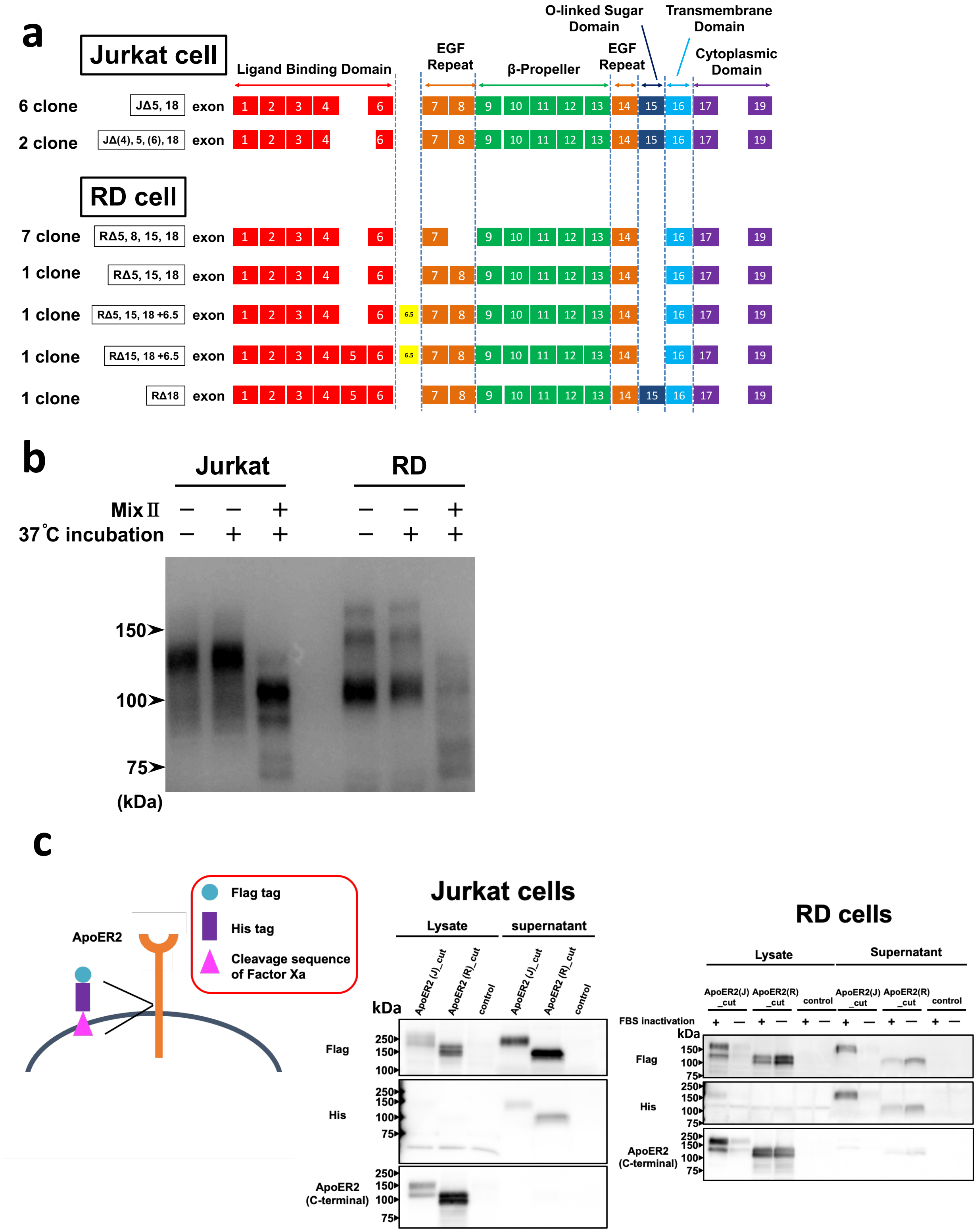

Supplementary Fig S4, Mizuno et al.,

Supplementary Fig. S4 | Cloning of the ApoER2 variants and the preparation of their soluble recombinant proteins. **a** ApoER2 variants expressed in Jurkat and RD cells. Total RNA was extracted from Jurkat and RD cells and cDNA was prepared as described in Materials and methods. Purified ApoER2 DNA was incorporated into pcDNA3.1, then transformed, and colonies were obtained. The plasmid DNA was purified and subjected to DNA sequence analysis. The figure is color-coded for each ApoER2 domain, and the numbers in the figure indicate the exon number. In the clones of JΔ(4),5,(6),18, RΔ5,15,18+6.5, and RΔ15,16+6.5, partial sequences of exons 4 and 5 and additional sequences between exons 6 and 7 were obtained as shown in each figure. **b** *N*- and *O*-glycosylation of ApoER2 expressed in Jurkat and RD cells. Whole cell lysate was prepared from each cell type and heated at 75°C for 10 min. Protein Deglycosylation Mix II was added and incubated at 25°C for 30 min. The reaction mixture was further incubated at 37°C for 1 h to prepare a whole sugar chain modified cleavage sample, which was subjected to western blotting using anti-ApoER2 Ab. **c** Preparation of soluble ApoER2 recombinant proteins. Left panel, on the 3' side of the transmembrane region (exon 16), the Flag and His tag sequences and Factor Xa cleavage sequence were inserted as described in the Materials and methods. The ApoER2(J)_cut and ApoER2(R)_cut plasmids were transfected into Jurkat and RD cells, respectively, and the conditioned medium was collected 48 h after transfection. The whole cell lysates and the conditioned medium were subjected to western blotting using Ab against each tag and ApoER2.

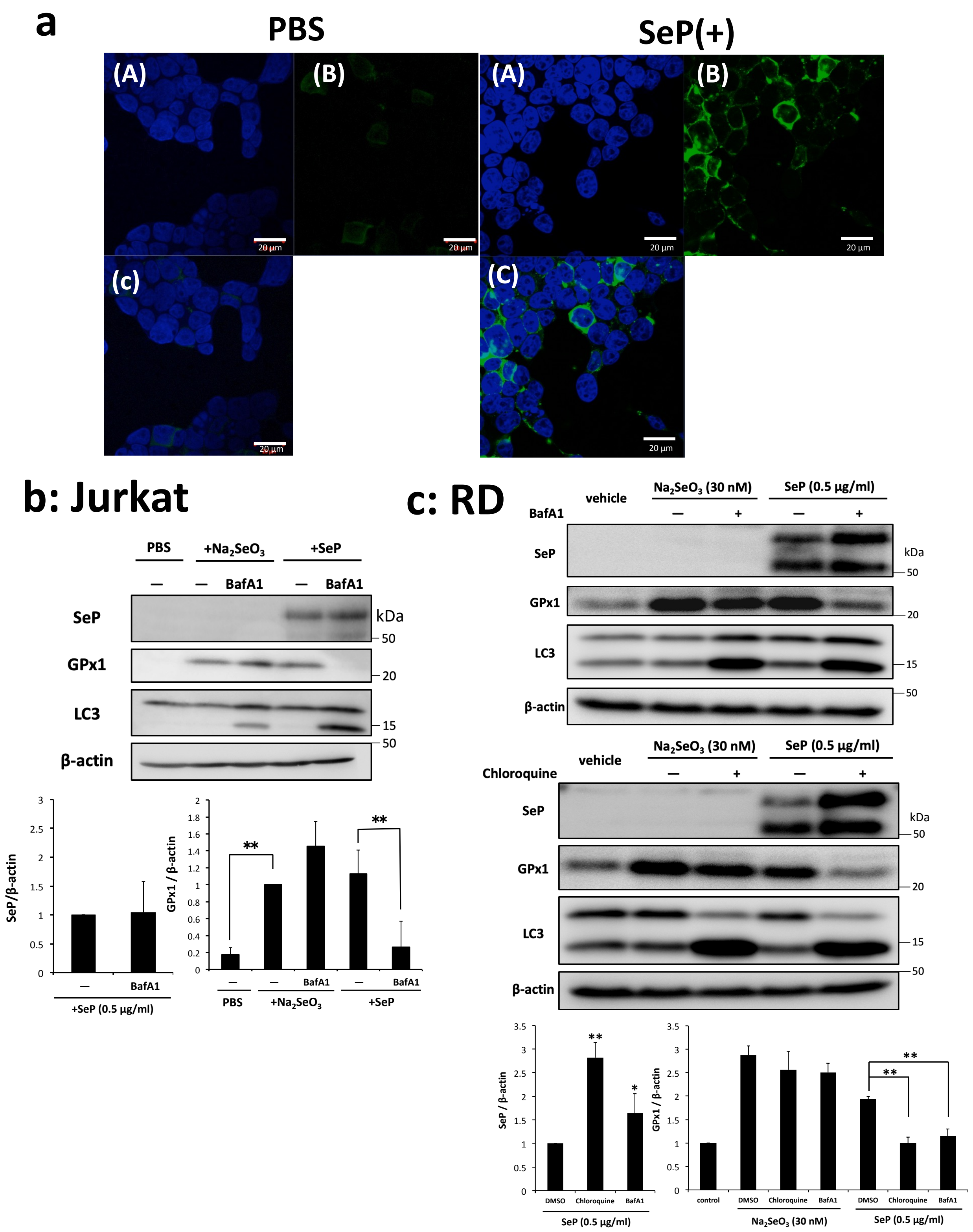

Supplementary Fig S5, Mizuno et al.,

Supplementary Fig. S5 | Evaluation of SeP uptake using immunostaining and effects of chloroquine on the Se-supply activity of SeP. **a** Immunohistochemical analysis of SeP-treated Jurkat cells (0.5 µg/ml SeP for 24 h) using anti-SeP Ab (green). Cell nuclei were stained using Hoechst (blue). Scale bars = 20 µm. **b, c** Effects of lysosomal inhibitors on cellular SeP and GPx1 levels in Jurkat and RD cells. The cells were cultured with 0.5 µg/ml SeP or 100 nM Na_2SeO_3 in the presence of 100 nM bafilomycin A1 (BafA1) or 100 µM chloroquine for 24 h. Then, whole cell lysates were subjected to western blotting (n = 3, means ± s.d.).

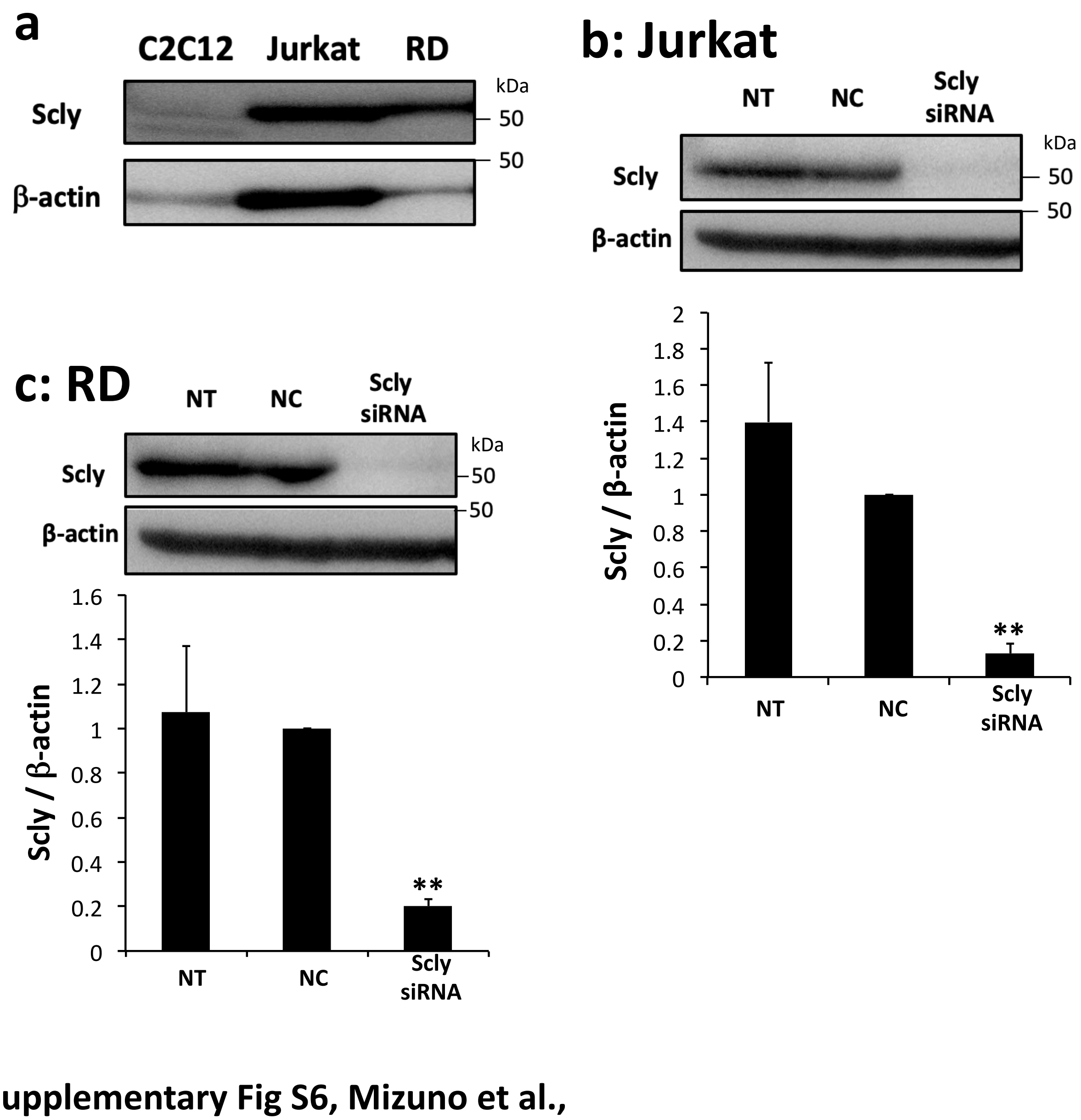

Supplementary Fig. S6 | Expression levels of selenocysteine lyase (Scly) and effects of its siRNA. **a** Western blotting of Scly in C2C12 myocytes, Jurkat cells, and RD cells. **b, c** Jurkat (b) and RD cells (c) were treated with Scly-siRNA or non-specific RNA (NC) and cultured for 72 h. The whole cell lysates were subjected to western blotting using anti-Scly Ab (n = 3, means ± s.d.). $^{**}P < 0.01$, Tukey ANOVA. NT: non transfection.

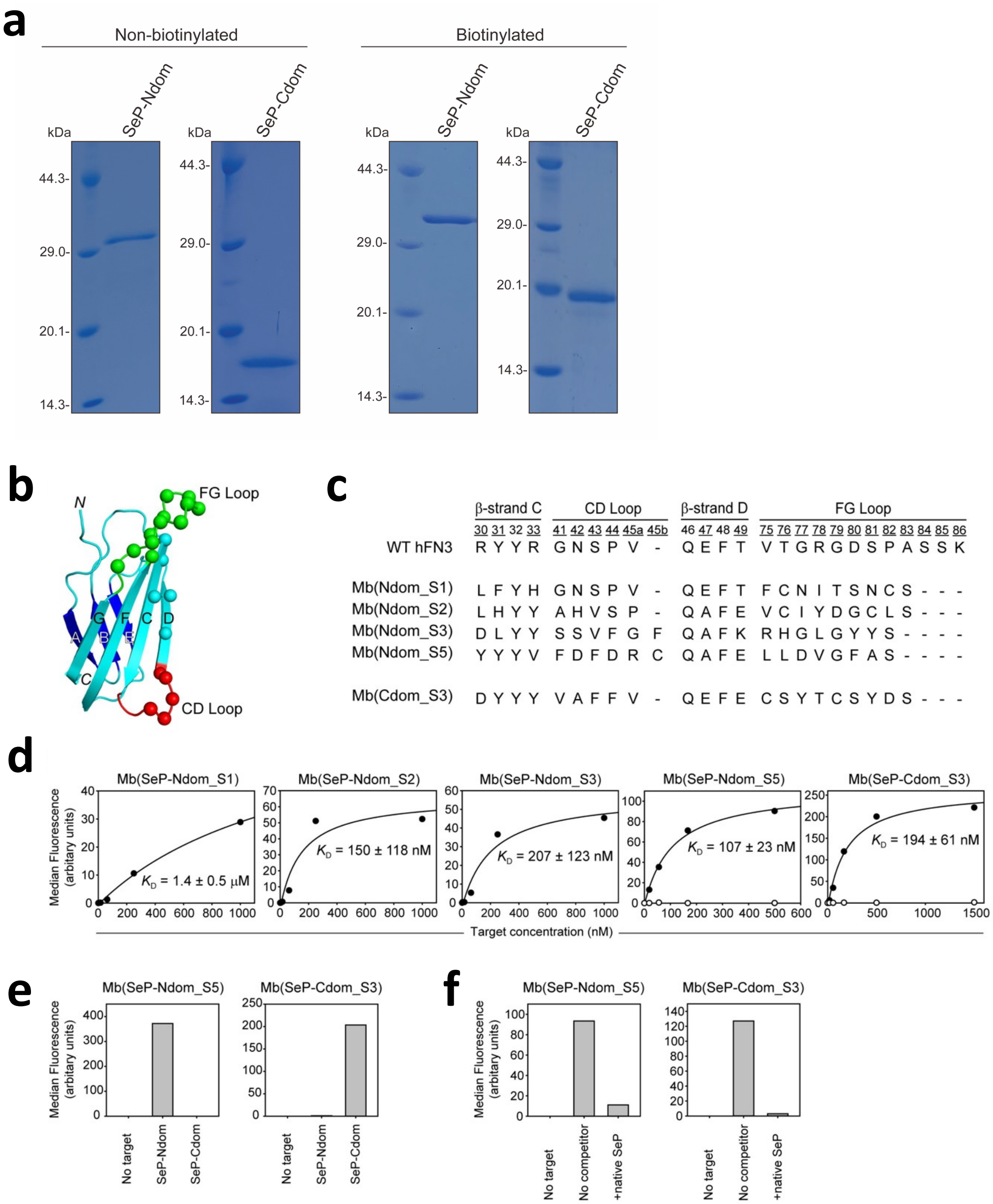

Supplementary Fig S7, Mizuno et al.,

Supplementary Fig. S7 | Characterization of the recombinant SeP-Ndom and SeP-Cdom and monobodies binding to SeP-Ndom and SeP-Cdom. **a** SDS-PAGE analysis of the recombinant SeP-Ndom and SeP-Cdom used in this study. The proteins (2.0–3.0 μg) were loaded onto 15% SDS-polyacrylamide gels and the gels were stained with Coomassie Brilliant Blue. Standard molecular weight markers (left lane in each gel) were used. **b** Schematic of the monobody scaffold, with the locations of diversified residues shown as spheres and strands, loops and termini labeled. **c** Amino acid sequences of monobodies, with the wild-type FN3 sequence as a reference. Residue numbers for diversified positions are underlined. The Ndom and Cdom series monobodies are directed to SeP-Ndom and SeP-Cdom, respectively. **d** Binding titration curves and the dissociation constants (K_D) of the monobodies measured using yeast surface display. The median fluorescence intensities of yeast cells displaying a monobody are plotted as a function of a respective cognate target concentration (filled circles). For Mb(Ndom_S5) and Mb(Cdom_S3), no binding to the denatured target was also tested to validate that these monobodies are truly conformationally specific (open circles). The denatured target was prepared by boiling the target protein for 15 min. The errors shown are the standard deviations from curve fitting of the 1:1 binding model. **e** Specificity analysis showing a lack of domain cross-reactivity of monobodies. Binding signals of Mb(Ndom_S5) and Mb(Cdom_S3) to their respective cognate and non-cognate targets at 500 nM are shown. **f** Competition binding experiments using native SeP as a competitor. Binding of monobodies indicated above each panel to SeP-Ndom or SeP-Cdom in the presence and absence of native SeP competitor is shown. Reduction in MFI values in the presence of native SeP compared to those in the absence of native SeP indicated that the selected monobodes bind native SeP. The SeP-Ndom and SeP-Cdom concentrations used were both 300 nM. The native SeP concentration used as a competitor was 2.67 μM.

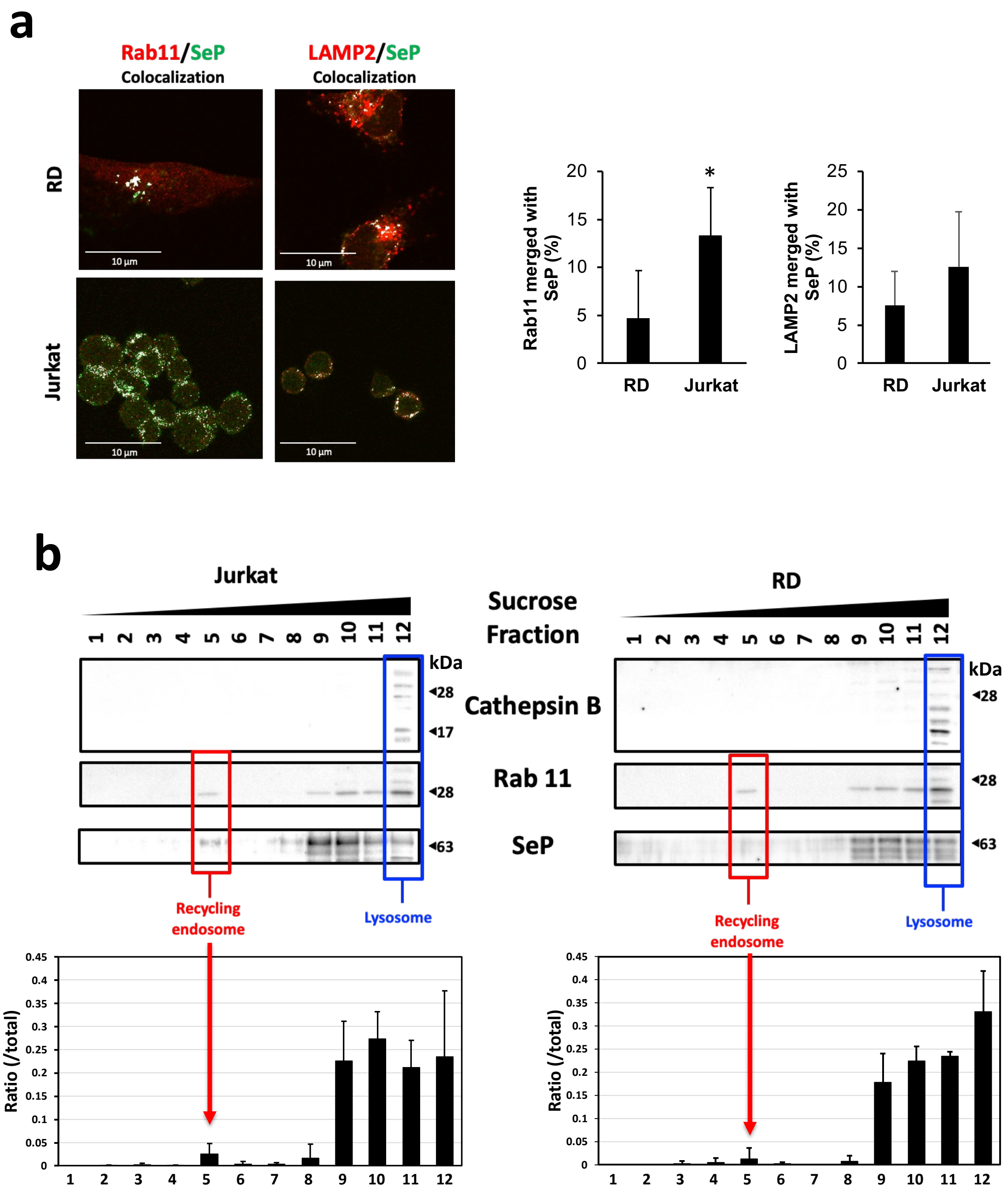

Supplementary Fig S8, Mizuno et al.,

Supplementary Fig. S8 | Analysis of the cellular localization of incorporated SeP. **a** Immunohistochemical analysis of SeP-treated RD cells (0.5 µg/ml SeP for 24 h) or SeP-treated Jurkat cells (0.5 µg/ml SeP for 24 h) using anti-SeP Ab (green) and anti-LAMP-2 Ab (red, indicative of lysosomes). SeP-treated cells were also stained using anti-Rab11 (red, indicative of

recycling endosomes). Cell nuclei were stained using Hoechst (blue). Scale bars = 10 μm. For pixel distribution analysis, the stained cells were analyzed by Fluoview software (n = 3, means ± s.d.). $*P < 0.05$, Student's *t* test. **b** Distribution of incorporated SeP in the subcellular fractions. Each cell type was treated with SeP for 6 h. Then, its homogenized product was separated using sucrose density gradient centrifugation, and equal-volume samples from each gradient fraction were subjected to western blotting using each marker protein. The band intensities of each protein were evaluated (n = 3, means ± s.d.). Fraction 5 had recycling endosomes. Cathepsin B: indicative of lysosomes.
